# Supplementary material for: Spatial variations and predictors of neonatal mortality among births to HIV-infected and non-infected mothers in rural Zambia
Source: PLOS Glob Public Health. 2025 Jul 1;5(7):e0003331. doi: 10.1371/journal.pgph.0003331 (PMC12212519; doi:10.1371/journal.pgph.0003331)
Supplement: Attachment — Submitted filename: Rewiewed Paper_PGPH-D-24-01165_R1.pdf [file pgph.0003331.s003.pdf]

PLOS Global Public Health

Spatial Variations and Predictors of Neonatal Mortality among HIV-Infected and Non-Infected Mothers in Rural Zambia

--Manuscript Draft--

Manuscript Number:	PGPH-D-24-01165R1
Article Type:	Research Article
Full Title:	Spatial Variations and Predictors of Neonatal Mortality among HIV-Infected and Non-Infected Mothers in Rural Zambia
Short Title:	Neonatal Mortality Among HIV-Infected and Non-Infected Mothers in Rural Zambia
Corresponding Author:	Samson Shumba, B.A., MSc., PGD-TM<br>University of Zambia<br>Lusaka, Lusaka ZAMBIA
Order of Authors:	Samson Shumba, B.A., MSc., PGD-TM
	Deborah Tembo, MSc, BSc, PGD, RN
	Miyanda Simwaka, MSc, MBCHb, BSc Hb
	Nedah Chikonde Musonda, MSc, BSc, RN
	Chipo Nkwemu, MSc, BSc, Dip, PGD
	Sebean Mayimbo, PhD, MSc, RM, RN
Keywords:	neonatal; Mortality; HIV; infected; rural; spatial
Abstract:	In Zambia, neonatal mortality still remains a noteworthy public health problem with a current rate of 27 deaths per 1000 and ranking 162 out of 195 countries globally. The study aimed to investigate the spatial variations and predictors of neonatal mortality in rural Zambia among HIV-infected and non-infected mothers. This study utilized the national-level data from the 2018 Zambia Demographic and Health Survey (ZDHS). Statistical analyses were conducted using the Rao – Scott Chi-square test to assess associations between neonatal mortality and categorical variables. Additionally, a multilevel mixed effect logistic regression model was used to examine predictors of neonatal mortality. Geospatial variations of neonatal mortality across Zambia's ten provinces were mapped using Quantum Geographical Information System (QGIS) version 3.34.1. Data analysis was performed using Stata version 14.2. The study findings indicate a higher mortality rate among HIV-infected mothers aged 45 to 49 (100%) compared to 95.21% among non-HIV-infected mothers. Neonatal mortality was notably elevated among neonates born to mothers with no education (40.78%) and those with HIV infection (54.56%). Employment status also influenced mortality rates, with employed non-HIV-infected mothers showing 36.35% mortality compared to 49.39% among unemployed HIV-infected mothers. Higher birth weights, particularly 4000 grams or higher, were associated with increased mortality for both HIV-infected (81.15%) and non-infected (21.00%) mothers. Multilevel logistic regression identified predictors of mortality, including maternal age (20-24 years), neonate’s sex (female), duration of breastfeeding, maternal education and number of children ever born. Geospatial analysis revealed Eastern and North-Western provinces as hotspots for neonatal mortality among HIV-infected mothers, while Muchinga was a hotspot for non-HIV-infected mothers. This study examined neonatal mortality among HIV-infected and non-infected mothers in rural Zambia, uncovering predictors such as maternal age, neonate sex, birthweight, maternal education, duration of breastfeeding and number of children ever had. Regional variations in mortality underscore the necessity for targeted interventions. Strengthening healthcare infrastructure, community outreach, healthcare worker training, maternal education, and addressing regional differences are crucial for improving maternal and child health and meeting Sustainable Development Goals targets.
Additional Information:	
**Question**	**Response**

**Financial Disclosure**<br><br>Enter a financial disclosure statement that describes the sources of funding for the work included in this submission and the role the funder(s) played. This includes grants and any commercial funding of the work or authors.<br><br>This statement will be typeset if the manuscript is accepted for publication.<br><br>*Please review the submission guidelines and the instructions link below for detailed requirements and guidance.*	The authors received no specific funding for this work.
**Competing Interests**<br><br>On behalf of all authors, disclose any competing interests that could be perceived to bias this work.<br><br>This statement will be typeset if the manuscript is accepted for publication.<br><br>*Please review the instructions link below and PLOS Global Public Health's competing interests policy to determine what information must be disclosed at submission.*	The authors have declared that no competing interests exists.
**Data Availability**<br><br>Before publication, Authors are required to make fully available and without restriction all data underlying their findings. Please see our PLOS Data Policy page for detailed information on this policy.<br><br>A **Data Availability Statement**, detailing where the data can be accessed, is required at first submission. Insert your	Authorization to utilize the data was secured from ICF Macro, accessible at https://dhsprogram.com/data, under the dataset titled ZMKR71FL.DTA and ZMAR71FL.DTA

Data Availability Statement in the box below.<br><br>Please see the data reporting section of our submission guidelines for instructions on what you need to include in your Data Availability Statement.<br><br>This statement will be typeset if the manuscript is accepted for publication.<br><br>*PLOS allows rare exemptions to address legal and ethical concerns. If you have legal or ethical restrictions, please use the box below to detail these in full sentences for the Journal team to consider.*	

Manuscript Click here to access/download;Manuscript;Manuscript.docx

Spatial Variations and Predictors of Neonatal Mortality among HIV-Infected and Non-Infected Mothers in Rural Zambia

Samson Shumba[1], Deborah Tembo[2], Miyanda Simwaka[3], Nedah Chikonde Musonda[4], Chipo Nkwemu[5] and Sebean Mayimbo[6]

1. Department of Epidemiology & Biostatistics, School of Public Health, University of Zambia, Lusaka, Zambia.
2. Department of Emergency Preparedness and Response. Zambia National Public Health Institute, Lusaka, Zambia.
3. Ministry of Health, Lusaka, Zambia.
4. Copperrose Zambia.
5. Centre for Infectious Disease Research in Zambia
6. Department of Midwifery, Women and Child Health, School of Nursing Sciences, University of Zambia, Lusaka, Zambia.

Corresponding Author

Samson Shumba

Samsonshumba1@gmail.com

Abstract

In Zambia, neonatal mortality still remains a noteworthy public health problem with a current rate of 27 deaths per 1000 and ranking 162 out of 195 countries globally. The study aimed to investigate the spatial variations and predictors of neonatal mortality in rural Zambia among HIV-infected and non-infected mothers. This study utilized the national-level data from the 2018 Zambia Demographic and Health Survey (ZDHS). Statistical analyses were conducted using the Rao – Scott Chi-square test to assess associations between neonatal mortality and categorical variables. Additionally, a multilevel mixed effect logistic regression model was used to examine predictors of neonatal mortality. Geospatial variations of neonatal mortality across Zambia's ten provinces were mapped using Quantum Geographical Information System (QGIS) version 3.34.1. Data analysis was performed using Stata version 14.2. The study findings indicate a higher mortality rate among HIV-infected mothers aged 45 to 49 (100%) compared to 95.21% among non-HIV-infected mothers. Neonatal mortality was notably elevated among neonates born to mothers with no education (40.78%) and those with HIV infection (54.56%). Employment status also influenced mortality rates, with employed non-HIV-infected mothers showing 36.35% mortality compared to 49.39% among unemployed HIV-infected mothers. Higher birth weights, particularly 4000 grams or higher, were associated with increased mortality for both HIV-infected (81.15%) and non-infected (21.00%) mothers. Multilevel logistic regression identified predictors of mortality, including maternal age (20-24 years), neonate's sex (female), duration of breastfeeding, maternal education and number of children ever born. Geospatial analysis revealed Eastern and North-Western provinces as hotspots for neonatal mortality among HIV-infected mothers, while Muchinga was a hotspot for non-HIV-infected mothers. This study examined neonatal mortality among HIV-infected and non-infected mothers in rural Zambia, uncovering predictors such as maternal age, neonate sex, birthweight, maternal education, duration of breastfeeding and number of children ever had. Regional variations in mortality underscore the necessity for targeted interventions. Strengthening healthcare infrastructure, community outreach, healthcare worker training, maternal education, and addressing regional differences are crucial for improving maternal and child health and meeting Sustainable Development Goals targets.

Keyword: spatial, neonatal, mortality, HIV

Introduction

Neonatal mortality is characterized by the death of a live-born infant, irrespective of the gestational age at birth, within the initial 28 completed days of life [1]. The initial month after birth represents a critical phase for child survival, marked by the unfortunate loss of 2.4 million newborns globally in the year 2020 [2]. During that same year, nearly 47% of all deaths in children under the age of five occurred during the neonatal period, which spans the first 28 days of life and 98% of the deaths occurred in low and middle-income countries (LMIC) [1-3]. This percentage reflects a notable increase from the 40% reported in 1990. The world has made impressive progress towards the survival of children since 1990. In 2020, the number of neonatal deaths declined from 5 million in 1990 to 2.4 million, nonetheless, the decline in neonatal mortality from 1990 to 2020 has been slower than that of the post-neonatal under-five mortality [1].

Sub-Saharan Africa bears the highest global neonatal mortality rate, reaching approximately 27 deaths per 1000 live births, and plays a significant role in global newborn fatalities, accounting for 43% of the total [4,5]. In close proximity, central and southern Asia report a neonatal mortality rate of 23 deaths per 1000 live births, contributing to 36% of global newborn deaths [1]. Moreover, a child born in Sub-Saharan Africa faces a staggering tenfold higher likelihood of dying in the first month than a child born in a high-income country. In Uganda a study revealed that the underlying causes of death are related to poor access and low utilization of health services during pregnancy and childbirth [6]. Furthermore, findings have revealed that more newborn deaths occur at home among the rural poor [7].

Zambia relative to other countries fairs poorly ranking 162 out of 195 countries globally in terms of neonatal mortality in Zambia [8]. According to Zambia Statistical Agency (2018) it indicated that neonatal mortality rate decreased from 37 to 27 neonatal deaths per 1000 live births between 2001 and 2018 [9]. Moreover, various studies have identified several factors correlated with heightened neonatal mortality rates in both the general population and facility-based settings. These include lower levels of maternal education [10-12], place of delivery and residence [13,14], maternal age [15,16], parity [17], gestational age [10,15], inadequate antenatal visits [18,19], newborn sex [13], newborn age [19], low birth weight [20], maternal and fetal complications [20], preterm birth [21], maternal BMI [22] and hypothermia [23]. Despite these findings, there remains a gap in understanding neonatal mortality and its associated risk factors, particularly within rural

communities, especially between HIV-infected and non-infected mothers in rural Zambia. Therefore, this study aimed to investigate the geospatial patterns and predictors of neonatal mortality among both HIV-infected and non-infected mothers in rural Zambia.

Conceptual Framework

We modified the conceptual framework of neonatal mortality using the analytical framework established by Mosley and Chen. Originally proposed in 1984, this analytical framework for child mortality was subsequently adapted to emphasize variations in infant and child mortality attributable to socio-economic differentials, bio-demographic factors, household environmental conditions, nutrient deficiencies, and community influences (Fig 1) [24].

Figure 1: Conceptual framework

Legend. Figure 1: Neonatal mortality conceptual framework

(Source: Mosley and Chen, 2003)

Methods

This study constitutes a secondary analysis of microdata utilizing national-level data sourced from the Zambia Demographic and Health Survey (ZDHS) program using the survey dataset in 2018. The ZDHS is a comprehensive, nationally representative household survey conducted by the Zambia Statistics Agency in collaboration with global partners, including ICF International and the United States Agency for International Development (USAID). The survey employs a two-stage sampling process, initially selecting enumeration areas (EAs) and subsequently households.

Dependent and independent variables

The primary outcome of interest in this study is neonatal mortality, defined as the death of infants within the first 28 days of life. The explanatory variables used are grouped into demographic, socio-economic, behavioral, and community-level factors to provide a holistic view of the determinants of neonatal mortality. Demographic variables include the mother's age (categorized as 15-19, 20-24, 25-29, 30-34, 35-39, 40-44, or 45-49 years), child's sex (male or female), and the

number of children ever born (birth order). These variables capture biological and family composition factors that influence neonatal outcomes. Socio-economic factors encompass maternal education (none, primary, secondary/tertiary), household wealth index (poorest, poorer, middle, richer, richest), marital status (married or not married), and employment status (employed or unemployed). These indicators reflect the mother's access to resources and social determinants of health. Behavioral factors focus on health-related practices, including the duration of breastfeeding (ever vs. never breastfed). These behaviors are critical in understanding neonatal care practices that affect survival during the first month of life. Community-level factors consist of the source of drinking water (improved or unimproved), place of delivery (home, private, or public health facility) and region. These variables capture the environmental and infrastructural context in which the mother and newborn reside, which can significantly affect health outcomes [25].

The selection of these variables bio-demographic, socio-economic, behavioral/environmental, and community-level factors aligns with existing research on neonatal mortality and reflects key determinants recognized by global health organizations such as the World Health Organization (WHO) and UNICEF. Each variable captures essential dimensions that influence maternal and child health outcomes, which are critical to informing policies and interventions.

Data Analysis

For descriptive analysis, frequencies and percentages were calculated for categorical variables to summarize the data. To assess the association between neonatal mortality (the outcome variable) and categorical predictors, the Rao–Scott Chi-square test was employed, accounting for the complex survey design. A multilevel mixed-effects logistic regression model was used to identify individual and community-level factors influencing neonatal mortality, recognizing the hierarchical structure of the data. The random factor in the study was cluster number coded as "v001".

The study followed an investigator-led approach for variable selection, drawing on a comprehensive review of relevant literature to ensure the inclusion of theoretically and empirically relevant predictors. Model selection was guided by the Akaike Information Criterion (AIC) and Bayesian Information Criterion (BIC), with the Intra-Class Correlation (ICC) used to quantify the

extent of variance explained at the community level. These measures ensured the identification of the most appropriate and parsimonious model. All analyses were performed using Stata version 14.2.

Furthermore, spatial variations of neonatal mortality across provinces in Zambia was done using the Quantum Geographic Information System (QGIS) version 3.34.1. The coordinate system utilized was the World Geographic System (WGS) 1984 Universal Transverse Mercator (UTM) Zone 36S.

Model Specification for Multilevel Mixed Effect Logistic Regression

$$logit\left(P(Y_{ij}=1)\right)=\beta_0+\sum_{k=1}^{k}\beta_k X_{ijk}+u_j$$

Where:

Logit is the log-odds function:

$$logit\left(P(Y_{ij}=1)\right)=\ln\left(\frac{P(Y_{ij}=1)}{1-P(Y_{ij}=1)}\right)$$

$u_j \sim N(0,\sigma_u^2)$ assumes that the cluster-level random effect follows a normal distribution with mean 0 and variance σ_u^2

Let:

- Y_{ij} = Outcome for individual i in cluster j (binary: $Y_{ij}=1$ if the event occurs, and $Y_{ij}=0$ otherwise).
- $X_{ij}=X_{ij1},X_{ij2},\ldots,X_{ijk}$ = vector of individual-level covariates
- β_0 = Fixed intercept (baseline log-odds of the outcome).
- β_k = Coefficients for individual-level predictors X_{ijk}
- u_j = Random intercept for cluster jjj, accounting for unobserved heterogeneity among clusters [26].

Intra-class Correlation ICC

The ICC quantifies the proportion of the total variance in the outcome attributable to differences between clusters:

$$ICC = \frac{\sigma_u^2}{\sigma_u^2 + \frac{\pi^2}{3}}$$

Here $\frac{\pi^2}{3}$ is the variance of the logistic distribution at the individual level [23].

Ethics statement

The methodologies used in the 2018 ZDHS, including biomarker measurement protocols, were ethically cleared by both the Inner City Fund (ICF) institutional review boards (IRBs) and the Tropical Diseases Research Centre (TDRC) in Zambia. Oral consent was obtained from respondents, with parental or guardian consent for adolescents under 18. Neonate information was collected from caregivers. Detailed information on the DHS consent process is available (https://www.dhsprogram.com/What-We-Do/Protecting-the-Privacy-of-DHS-Survey-Respondents.cfm). Authorization to use ZDHS data was granted by ICF Macro, and the dataset can be accessed (https://www.dhsprogram.com/data). The user strictly adhered to confidentiality guidelines, ensuring the anonymity of respondents.

Results

Participation in the survey was limited to neonates aged one month from selected households of women who had consented to take part in the research. A total of 2015 participants were enrolled into the study comprising neonates from HIV-infected and non-infected mothers (Fig 2).

Figure 2: Description of sample derivation criteria

~~**Legend. Figure 2: Description of sample derivation criteria**~~

The map was generated using PowerPoint 2016. (Source: author generated). Dataset source: ZDHS women dataset accessible at: (https://www.dhsprogram.com/data).

The data presented in Table 1 provides an overview of neonatal mortality in rural settings among mothers infected with HIV and those not infected with HIV, drawing from the 2018 Zambia Demographic and Health Survey dataset. The findings reveal distinct patterns in neonatal mortality across various background characteristics, including maternal age, education level, employment status, neonate gender, birth order, birthweight, place of delivery, and HIV status. Notably, neonatal mortality increases significantly with maternal age ($p<0.0001$), with the highest mortality

observed in the age group 45 to 49 (100%) among HIV-infected mothers compared to 95.21% in non-HIV infected mothers.

Furthermore, mortality rates were higher among HIV-infected mothers (54.56%) compared to non-HIV-infected mothers without formal education (40.78%). Overall, neonatal mortality stood at 41.13% among mothers (both HIV and non-HIV-infected) with no formal education. Neonatal mortality was significantly higher among HIV-infected mothers who were not working (49.39%), in contrast to non-HIV-infected mothers, where high mortality was observed among those who were employed (36.36%).The neonatal mortality rate was high at 37.17% among neonates whose mothers (both HIV infected and non-infected mothers) were reported to be working compared to those who were not working (26.14%). Neonatal mortality was also higher among males in both HIV-infected and non-infected mothers (52.99% and 33.24%, respectively). Moreover, neonatal mortality was consistently higher among neonates with a birth order of 7 or higher among HIV-infected and non-infected mothers in rural Zambia (81.15% and 61.82%), with overall mortality also reported high among those of the 7th or higher birth order (63.73%).

Additionally, neonatal mortality was reported higher among neonates born with a weight of 4000 grams or higher among HIV-infected and non-infected mothers (81.15% and 21.00%, respectively). Overall, neonatal mortality was higher among mothers infected with HIV (51.48%) compared to those not infected (29.89%).

Table 1: Key attributes of neonatal mortality in rural setting of Zambia in 2018

Variables	HIV – Infected (n=172)		Non-HIV infected (n=1,833)		Overall (n=2,005)	
	Alive	Dead	Alive	Dead	Alive	Dead
**Age of mother**	<0.0001*		0.0001*		<0.0001*	
15 - 19	2 (100)	0 (0.00)	247 (94.28)	15 (5.72)	249 (94.54)	15 (94.54)
20 - 24	23 (70.71)	9 (29.29)	341 (83.58)	67 (16.42)	372 (82.73)	78 (17.27)
25 – 29	11 (59.26)	8 (40.74)	230 (77.87)	65 (22.13)	248 (76.61)	76 (23.39)
30 – 34	22 (70.25)	9 (29.75)	182 (69.47)	79 (45.30)	214 (70.11)	91 (29.89)
35 – 39	18 (59.28)	12 (40.72)	117 (54.70)	97 (45.30)	136 (54.73)	113 (45.27)
40 – 44	8 (26.76)	21 (73.24)	60 (38.01)	98 (61.99)	68 (35.63)	122 (64.37)
45 - 49	0 (0.00)	18 (100.00)	4 (4.79)	81 (95.21)	4 (3.94)	99 (96.06)
**Education level of the mother**	0.584		<0.0001*		<0.0001*	
No Education	12 (45.44)	14 (54.56)	127 (59.22)	88 (40.78)	148 (58.87)	103 (41.13)
Primary	44 (48.62)	46 (51.38)	723 (68.56)	332 (31.44)	786 (67.10)	386 (32.90)
Secondary	26 (61.51)	16 (38.49)	317 (79.79)	80 (20.21)	351 (77.80)	100 (22.20)
Higher	1 (50.64)	1 (49.36)	13 (77.54)	4 (22.46)	15 (75.30)	5 (24.70)
**Marital status**	0.6842		0.1775		0.3384	
Not married	32 (54.13)	27 (45.87)	279 (73.61)	100 (26.39)	319 (70.94)	131 (29.06)
Married	51 (49.94)	50 (50.07)	902 (69.09)	404 (30.91)	981 (67.93)	463 (32.07)
**Wealth Index of the mother**	0.92		0.0821		0.1169	
Poorest	25 (51.17)	24 (48.83)	456 (68.68)	208 (31.32)	501 (67.91)	237 (32.09)
Poorer	27 (56.59)	21 (43.41)	407 (75.12)	135 (24.88)	440 (73.27)	160 (26.73)
Middle	21 (50.44)	20 (49.56)	224 (64.91)	121 (35.09)	256 (63.89)	145 (36.11)
Richer	7 (44.18)	8 (55.82)	66 (65.34)	35 (34.66)	73 (62.62)	43 (37.38)
Richest	3 (41.28)	5 (58.72)	27 (86.17)	4 (13.83)	32 (77.92)	9 (22.08)
**Mother currently working**	0.8562		0.0001*		0.0002*	
No	41 (50.61)	40 (49.39)	678 (75.71)	218 (24.29)	739 (73.86)	261 (26.14)
Yes	41 (52.37)	38 (47.63)	503 (63.74)	286 (36.26)	562 (62.83)	333 (37.17)

**Sex of child**	0.1504		0.0048*		0.0003*	
Male	45 (47.01)	51 (52.99)	599 (66.76)	298 (33.24)	658 (64.77)	358 (35.23)
Female	37 (58.24)	27 (41.76)	582 (73.93)	205 (26.07)	642 (73.13)	236 (26.87)
**Birth Order**	0.0004*		<0.0001		<0.0001*	
1-3rd birth order	37 (63.39)	21 (36.61)	682 (86.41)	107 (13.59)	741 (85.02)	130 (14.98)
4-6th birth order	39 (61.11)	25 (38.89)	338 (71.39)	136 (28.61)	390 (70.17)	166 (29.83)
7th + order	7 (18.85)	32 (81.15)	161 (38.18)	261 (61.82)	169 (36.27)	298 (63.73)
**Place of Delivery**	0.3923		0.015*		0.0187*	
Home	7 (81.33)	1 (18.67)	164 (83.53)	32 (16.47)	179 (83.87)	34 (16.13)
Private health facility	8 (100.00)	0 (0.00)	84 (95.45)	4 (4.55)	1003 (89.13)	122 (10.87)
Government	65 (79.26)	17 (20.74)	912 (89.91)	102 (10.09)	92 (95.83)	4 (4.17)
Unspecified	4 (100.00)	0 (0.00)	21 (82.61)	4 (17.39)	27 (81.92)	36 (18.08)
**Birth weight**	0.0004*		<0.0001*		<0.0001*	
Less than 2500	37 (63.39)	21 (36.61)	72 (80.83)	17 (19.17)	82 (80.71)	20 (19.29)
2500 - 3999	39 (61.11)	25 (38.89)	789 95.05)	41 (4.95)	862 (94.27)	52 (5.73)
4000+	7 (18.85)	32 (81.15)	319 (79.00)	85 (21.00)	357 (79.02)	95 (20.98)
**ANC visits**	0.7065		0.6983		0.8741	
<8 ANC visits	81 (86.97)	12 (13.02)	1142 (95.19)	58 (4.81)	1257 (94.59)	72 (5.41)
8+ ANC visits	1 (100.00)	0 (0.00)	17 (92.95)	1 (7.05)	19 (93.68)	1 (6.32)
**HIV Results**					<0.0001*	
HIV negative	NA	NA	NA	NA	1180.75	503 (29.89)
HIV positive	NA	NA	NA	NA	83 (51.48)	78 (48.52)
**Duration of breastfeeding**	<0.0001		<0.0001		<0.0001	
Ever breastfed	4 (25.03)	13 (74.97)	45 (38.37)	72 (61.63)	49 (36.66)	85 (63.34)
Never breastfed	1 (9.32)	6 (90.68)	5 (6.82)	71 (93.18)	6 (7.01)	77 (92.99)
Still breastfeeding	78 (100.00)	0 (0.00)	1131 (100.00)	0 (0.00)	1209 (100.00)	0 (0.00)
**Children ever born**	0.008		<0.0001		<0.0001	
Zero to two	24 (68.46)	11 (31.54)	530 (89.77)	60 (10.23)	71 (11.41)	554 (88.59)
three to four	26 (57.09)	20 (42.91)	279 (77.62)	80 (22.38)	100 (24.71)	305 (75.29)
five to six	25 (62.64)	15 (37.36)	211 (67.38)	102 (32.62)	117 (33.16)	236 (66.84)
seven plus	7 (18.85)	32 (81.15)	161 (38.18)	261 (61.82)	293 (63.49)	168 (36.51)

**Source of drinking water**	0.4797		0.4745		0.696	
Improved	61 (53.13)	54 (46.87)	625 (71.06)	255 (29.94)	309 (31.02)	686 (68.98)
Unimproved	21 (47.23)	24 (52.77)	556 (69.07)	249 (30.93	273 (32.09)	577 (67.91)
**Type of toilet facility**	0.0727		0.6089		0.3667	
Improved	40(60.79)	25 (39.21)	428 (71.01)	175 (28.99)	467 (70.02)	200 (29.98)
Unimproved	43 (45.22)	53 (54.78)	753 (69.61)	329 (30.39)	796 (67.62)	381 (32.38)
**Type of cooking fuel**	0.0125		0.029		0.0056	
safe	4 (100.00)	0 (0.00)	21 (88.17)	3 (11.83)	25 (88.63)	3 (11.37)
unsafe	79 (50.34)	78 (49.66)	1160 (69.85)	501 (30.15)	1239 (68.18)	578 (31.82)

All tests used the Rao – Scott Chi-square test, ANC: Antenatal Care, NA: Not Applicable

Geospatial analysis of neonatal mortality across rural Zambia among HIV-infected mothers

The study examined the geospatial patterns of neonatal mortality among mothers infected with HIV in rural Zambia. It revealed that North-western and Eastern provinces exhibited a high proportion of neonatal mortality, followed by Western and Central provinces. In contrast, Southern and Luapula provinces reported the lowest rates of neonatal mortality among HIV-infected mothers (Fig 3).

Legend. Figure 3: Shows the proportion of neonatal mortality among HIV infected mothers in rural Zambia (2018)

The map was generated using QGIS version 3.34.1. (Source: author generated). Source of the shapefile: https://data.humdata.org/dataset/cod-ab-zmb?#. Dataset source: ZDHS women dataset accessible at: (https://www.dhsprogram.com/data).

Geospatial analysis of neonatal mortality across rural Zambia among non-HIV infected mothers (2018)

The study revealed geospatial patterns of neonatal mortality among non-HIV-infected mothers in rural Zambia. Specifically, Muchinga provinces emerged as a frontrunner in terms of neonatal mortality rates, with Western, Copperbelt and Lusaka provinces following closely behind. Conversely, North-western, Luapula and Central provinces exhibited the lowest rates of neonatal mortality as shown in Fig 4.

Legend. Figure 4: Shows the proportion of neonatal mortality among non HIV-infected mothers in rural Zambia (2018)

The map was generated using QGIS version 3.34.1. (Source: author generated). Source of the shapefile: https://data.humdata.org/dataset/cod-ab-zmb?#. Dataset source: ZDHS women dataset accessible at: (https://www.dhsprogram.com/data).

Multilevel mixed effect logistic regression of neonatal mortality among mothers in rural Zambia

The adjusted model (Table 2), reveals several significant factors associated with neonatal mortality, controlling for other variables. Female neonates had a 65% lower chance of dying compared to males (AOR: 0.35, 95% CI: 0.15–0.84, p = 0.019), holding other factors constant. Births in public health facilities posed a significantly higher risk compared to home deliveries (AOR: 6.93, 95% CI: 1.58–30.45, p = 0.010). Similarly, neonates born to mothers with primary education had 88% lower odds of mortality than those whose mothers had no formal education (AOR: 0.12, 95% CI: 0.02–0.79, p = 0.028). Neonates who were never breastfed faced much higher mortality risks than those who were breastfed (AOR: 6.15, 95% CI: 1.89–19.98, p = 0.003). Regional variations were also apparent, as mothers from North Western Province had a significantly lower likelihood of neonatal mortality compared to those from Central Province (AOR: 0.14, 95% CI: 0.02–0.94, p = 0.043), indicating potential disparities in healthcare access or quality across regions.

Neonates born to HIV-positive mothers have almost five times higher odds of death compared to those born to HIV-negative mothers (AOR: 4.83, 95% CI: 0.90–25.91, p = 0.066), however we could not rule out chance finding. While this suggests that neonates born to HIV-positive mothers are nearly five times more likely to die, the wide confidence interval and p-value above 0.05 indicate that this result may lack precision and certainty. Other variables, such as maternal age, employment, and birthweight, did not show statistically significant associations with neonatal mortality when holding other factors constant. For example, mothers aged 35–39 exhibited lower odds of neonatal mortality (AOR: 0.12, 95% CI: 0.01–1.14, p = 0.065) than younger mothers aged 15–19, but the p-value did not meet the 0.05 threshold for significance. Similarly, neonates born to HIV-positive mothers had higher odds of mortality (AOR: 4.83, 95% CI: 0.90–25.91, p = 0.066).

The findings from the study revealed an intra-class correlation coefficient (ICC) of 3.81×10^{-33} and an intercept of 1.25×10^{-32} for the random effect associated with cluster number. These values suggest that almost all the variability in neonatal mortality is not attributable to differences between clusters (enumeration areas) but rather to individual-level factors. In practical terms, this indicates a minimal clustering effect, meaning that the neonatal mortality rates across clusters are

relatively homogeneous, and most of the differences arise from individual characteristics (e.g., maternal education, breastfeeding, or HIV status). Additionally, multicolinearity diagnostics were performed, with all variables in the adjusted model showing Variance Inflation Factors (VIFs) below 5, confirming that multicolinearity was not a concern. This ensures the reliability of the model's estimates by indicating that the independent variables are not excessively correlated with one another.

Table 2: Multilevel mixed effect logistic regression of neonatal mortality of HIV-infected and non-infected mothers in rural Zambia

Variables	COR (95% CI)	P-value	AOR (95% CI)	P-value
**Mother's age**				
15 - 19	Ref (1)		Ref (1)	
20 - 24	2.90 (1.70 - 4.94)	<0.001	3.49 (0.86 - 14.22)	0.081
25 -29	4.71 (2.75 - 8.07)	<0.001	1.63 (0.30 - 8.80)	0.570
30 - 34	7.34 (4.31 - 12.49)	<0.001	2.02 (0.32 - 12.70)	0.452
35 - 39	10.69 (6.21 - 18.41)	<0.001	0.12 (0.01 - 1.14)	0.065
40 -44	29.23 (16.51 - 51.77)	<0.001	3.88 (0.29 - 52.72)	0.309
45 - 49	399.36 (140.72 - 1133.39)	<0.001		
**Wealth Index**				
Poorest	Ref (1)		Ref (1)	
Poorer	0.81 (0.62 - 1.05)	0.105	2.48 (0.80 - 7.68)	0.115
Middle	1.16 (0.87 - 1.55)	0.323	2.78 (0.77 - 10.04)	0.118
Richer	0.99 (0.61- 1.59)	0.959	9.31 (0.78 - 111.08)	0.078
Richest	0.72 (0.34 - 1.50)	0.386	8.19 (0.35- 189.69)	0.189
**Sex of child**				
Male	Ref (1)		Ref (1)	
Female	0.70 (0.57 - 0.87)	0.001	0.35 (0.15 - 0.84)	0.019*
**Place of Delivery**				
Home	Ref (1)		Ref (1)	
Private	0.19 (0.06 - 0.56)	0.003	0.50 (0.02 - 11.73)	0.667
Public	0.54 (0.36 - 0.82)	0.003	6.93 (1.58 - 30.45)	0.010*
**Education**				
None	Ref (1)		Ref (1)	
Primary	0.62 (0.46 - 0.84)	0.002	0.12 (0.02 - 0.79)	0.028*
Secondary/tertiary	0.33 (0.23 - 0.47)	<0.001	0.20 (0.02 - 1.72)	0.143
**Marital status**				
Not married	Ref (1)		Ref (1)	
Married	1.27 (0.98 - 1.63)	0.068	2.39 (0.79 - 7.19)	0.122
**Employment**				
No	Ref (1)		Ref (1)	
Yes	1.90 (1.53 - 2.37)	<0.001	1.57 (0.63 - 3.92)	0.335

**Birth order**				
0 - 3	Ref (1)		Ref (1)	
4 - 6	2.91 (2.22 - 3.80)	<0.001		
7 +	10.66 (0.02 - 14.16)	<0.001	1.10 (0.14 - 8.69)	0.926
**Birthweight**				
<2500	Ref (1)		Ref (1)	
2500 - 3999	0.20 (0.11 - 0.36)	<0.001	0.42 (0.09 - 1.90)	0.258
4000 - 9999	1.19 (0.68 - 2.07)	0.55	2.38 (0.40 - 14.22)	0.343
**HIV status**				
Negative	Ref (1)		Ref (1)	
Positive	2.42 (1.69 - 3.47)	<0.001	4.83 (0.90 - 25.91)	0.066
**Toilet**				
Improved	Ref (1)		Ref (1)	
Unimproved	1.09 (0.86 - 1.39)	0.466	1.00 (0.34 - 2.96)	0.999
**Duration of breastfeeding**				
Ever breastfed,	Ref (1)		Ref (1)	
Never breastfed	6.08 (2.69 - 13.74)	<0.001	6.15 (1.89 - 19.98)	0.003*
**Source of drinking water**				
Improved	Ref (1)		Ref (1)	
Unimproved	1.00 (0.80 - 1.26)	0.967	0.95 (0.37 - 2.44)	0.909
**Region**				
Central	Ref (1)		Ref (1)	
Copperbelt	1.48 (0.77 - 2.85)	0.242	2.78 (0.15 - 51.91)	0.493
Eastern	1.32 (0.79 - 2.22)	0.294	1.63 (0.23 - 11.59)	0.623
Luapula	1.28 (0.74 - 2.19)	0.378	1.92 (0.27 - 13.80)	0.519
Lusaka	0.97 (0.48 - 1.95)	0.923	0.06 (0.003 - 1.26)	0.071
Muchinga	2.23 (1.30 - 3.82)	0.004	2.67 (0.46 - 15.68)	0.272
Northern	0.79 (0.45 - 1.40)	0.422	1.65 (0.22 - 12.48)	0.630
North Western	0.63 (0.34 - 1.19)	0.155	0.14 (0.02 - 0.94)	0.043*
Southern	0.99 (0.56 - 1.73)	0.965	2.10 (0.31 - 14.39)	0.449
Western	1.85 (1.08 - 3.17)	0.026	0.43 (0.08 - 2.41)	0.335

COR: Crude Odds Ratio AOR: Adjusted Odds Ratio

Discussion

This study investigated the spatial variations and predictors of neonatal mortality among HIV-infected and non-infected mothers in rural Zambia, utilizing the 2018 Zambia Demographic Health Survey (ZDHS) dataset. Our analysis revealed distinctive patterns in neonatal mortality across various demographic factors shedding light on the intricate interplay of these factors influencing neonatal mortality survival and highlighting regional disparities in neonatal mortality rates in rural Zambia among HIV infected and non-infected mothers. Notably, neonatal mortality exhibited a significant association in the baseline characteristics with an increase in maternal age, reaching its

peak in the 45 to 49 age group, accounting for 100% among HIV infected mothers compared to 95.21% in non- HIV-infected mothers.

However, utilizing multilevel logistic regression, our study found that only women aged 35 to 39 years had lower odds of experiencing neonatal mortality compared to younger mothers aged 15 to 19 years, regardless of their HIV status albeit that maternal age was not statistically significant. This finding is consistent with evidence showing that maternal age can significantly influence neonatal outcomes, with younger and older maternal age groups carrying unique risks. While adolescent mothers (aged 15–19) are often considered high-risk due to socio-economic vulnerabilities and limited access to healthcare, adulthood presents its own set of challenges, such as physiological and emotional stressors, which can adversely affect neonatal health [27-29].

Several studies highlight that maternal age influences birth outcomes through various mechanisms. Young mothers may have better recovery rates from pregnancy-related stress, while adult women might face life transitions (e.g., balancing education, employment, or childbearing) that could limit their access to quality antenatal care. Moreover, socio-cultural expectations, especially in low-resource settings, may push women to have more children or space them closely, increasing the risk of adverse neonatal outcomes [30].

Furthermore the study examined the relationship between maternal HIV status and neonatal mortality in Zambia, utilizing an adjusted model to account for confounding factors. Initial unadjusted analysis indicated a higher risk of mortality for neonates born to HIV-positive mothers compared to those born to HIV-negative mothers. However, the adjusted model revealed that this association was not statistically significant. These findings align with existing literature, which suggests that while maternal HIV infection is associated with increased neonatal mortality risk, the relationship is complex and influenced by various factors. Research has demonstrated that access to quality healthcare and antiretroviral therapy (ART) plays a crucial role in mitigating risks associated with maternal HIV. ART has been shown to significantly reduce vertical transmission of HIV and improve maternal health outcomes, thus potentially lowering neonatal mortality rates among HIV-positive mothers [31,32].

Additionally, socio-economic conditions can greatly affect healthcare access and adherence to ART, further complicating the relationship between maternal HIV status and neonatal outcomes

[33,34]. The uncertainty in the adjusted findings from this study highlights the variability in healthcare access and ART adherence among HIV-positive mothers, which is supported by literature indicating disparities in healthcare utilization and treatment adherence across different socio-economic context [35-37]. This underscores the need for comprehensive interventions that not only address HIV but also improve overall maternal healthcare and socio-economic conditions to enhance neonatal outcomes.

The geospatial distribution of neonatal mortality among HIV-infected and non-infected mothers in rural Zambia encompassed all 10 provinces of the country. The analysis revealed significant spatial disparities in neonatal mortality rates, with notable hotspots identified in the North-Western and Eastern provinces for HIV-infected mothers, and in Muchinga province for non-HIV-infected mothers. This discrepancy in neonatal mortality rates across provinces may stem from multiple factors, including disparities in access to healthcare services, variations in socio-economic conditions, differences in maternal education levels, prevalence of diseases, and cultural practices related to childbirth and infant care [32,33,35,38] . The data emphasizes the dynamic nature of neonatal mortality proportions across Zambia's provinces during the specified period. Recognizing the complexities that contribute to neonatal mortality is crucial for designing targeted interventions aimed at reducing mortality rates and improving maternal and child health outcomes nationwide [31,36].

Our multilevel model revealed that female neonates exhibited a lower likelihood of neonatal mortality, indicating that male neonates were more vulnerable to mortality within the first month of life compared to their female counterparts. These findings align with prior research indicating a higher mortality rate among boys compared to girls [39,40]. Furthermore the findings indicate that maternal education significantly influences neonatal mortality, with primary education serving as a protective factor. Mothers with primary education are associated with 88% lower odds of neonatal death compared to those with no education. While secondary or tertiary education shows a similar protective trend (AOR: 0.20, 95% CI: 0.02–1.72), it does not reach statistical significance ($p = 0.143$). These results suggest that even basic education equips mothers with essential knowledge to improve neonatal health outcomes, reinforcing the need to promote educational access among women in rural areas.

Recent findings highlight the strong impact of maternal education on neonatal and child mortality. A systematic review and meta-analysis published in the lancet found a clear dose-response relationship: mothers with 12 years of education (equivalent to secondary school completion) were associated with a 31% reduction in under-5 mortality compared to those with no education. Additionally, each additional year of maternal schooling reduced child mortality by approximately 3%. The study emphasizes that maternal education remains a stronger predictor of child survival compared to paternal education, underscoring its transgenerational benefits for child health [38] These findings align with recommendations in the WHO's 2023 progress report on maternal and newborn health, which calls for investments in education as part of strategies to reduce neonatal mortality. The report highlights the need for integrated efforts, including better healthcare access and education, to meet global health goals [41].

Furthermore our study emphasizes the multifaceted nature of factors influencing neonatal mortality, including maternal age, neonate sex, maternal HIV status, and duration of breastfeeding, place of delivery and Region (North Western province). This intricate web of variables highlights the need for a shift in focus within current mother and child health programs to incorporate activities that empower women to make informed decisions regarding the timing of childbirth, monitor their HIV status, duration of breastfeeding and consider the birthweight and sex of their child. Additionally, a systematic review and meta-analysis conducted in sub-Saharan Africa revealed that rural residence significantly contributes to high neonatal mortality rates [42]. Our study's novel findings offer valuable insights previously unexplored, illuminating the intricate landscape of neonatal mortality in rural Zambia. Recognizing the distinct patterns of neonatal mortality between HIV-infected and non-infected mothers is crucial for implementing tailored interventions that enhance maternal and child health outcomes within these communities.

Study strength and study Limitations

This study benefits from the utilization of national data, offering a representative sample of neonates, thus enabling generalizability to this specific demographic. However, certain limitations must be acknowledged. The study relies on the 2018 Zambia Demographic and Health Survey (ZDHS) dataset, employing a cross-sectional design, which indicates correlation rather than causation between the outcome and individual or contextual factors. Additionally, the utilization

of contextual and community-level factors from the ZDHS may not fully capture the community experience. Moreover, potential recall bias exists, with mothers possibly misreporting crucial information like their child's age at death, birth order, and birth weight. To address this, rigorous data cleaning procedures were implemented to identify and rectify inconsistencies or outliers.

Policy recommendation

To effectively address the disparities in neonatal mortality rates among HIV-infected and non-infected mothers in rural Zambia, it is essential to implement a multifaceted approach that focuses on improving access to quality healthcare services, particularly in the identified hotspot regions of North-Western, Eastern, and Muchinga provinces. This can be achieved by enhancing healthcare infrastructure and resources, ensuring the availability of essential maternal and child health services, and increasing the reach of antiretroviral therapy (ART) programs for HIV-positive mothers. Additionally, targeted educational campaigns should be developed to raise awareness about safe childbirth practices, infant care, and the importance of maternal health, particularly in communities with low maternal education levels. Collaborating with local stakeholders, including traditional leaders and community health workers, can foster culturally sensitive interventions that resonate with local practices and beliefs. Furthermore, addressing socio-economic determinants, such as poverty and access to education, will be crucial in creating an enabling environment for improving maternal and child health outcomes across all provinces.

Conclusion

In conclusion, this study highlights significant geospatial variations in neonatal mortality rates among HIV-infected and non-infected mothers across rural Zambia, emphasizing the need for targeted interventions to address these disparities. The findings reveal that regional differences in access to healthcare, socio-economic conditions, maternal education levels, and cultural practices significantly influence neonatal outcomes. Addressing these multifaceted factors is crucial for improving maternal and child health in Zambia. By enhancing healthcare access, increasing the availability of antiretroviral therapy, and implementing culturally sensitive educational campaigns, stakeholders can work collaboratively to reduce neonatal mortality rates. Ultimately, a comprehensive approach that considers the unique challenges faced by different provinces will be

essential for achieving equitable health outcomes and promoting the well-being of mothers and their infants throughout the country.

Acknowledgement

Many thanks to the Zambia Statistical Agency (ZSA) and the DHS program for granting permission to utilize the 2018 ZDHS.

Dedication

This paper is dedicated, with love and reverence, to the cherished memory of Wise Mwila Katumbo.

Reference

1. Newborn Mortality (who.int) https://www.who.int/news-room/fact-sheets/detail/levels-and-trends-in-child-mortality-report-2021
2. Hug L, Sharrow D, Zhong K, You D. Levels & trends in child mortality. New York, USA: United Nations Children's Fund. 2017:1-40.
3. UN Inter-Agency Group for Child Mortality Estimation. UNICEF. WHO. Levels & Trends in Child Mortality Estimation Child Mortality. UNICEF; New York, NY, USA: 2020.
4. Akombi BJ, Renzaho AM. Perinatal mortality in sub-Saharan Africa: a meta-analysis of demographic and health surveys. Annals of global health. 2019;85(1).
5. Tekelab T, Chojenta C, Smith R, Loxton D. The impact of antenatal care on neonatal mortality in sub-Saharan Africa: A systematic review and meta-analysis. PloS one. 2019 Sep 13;14(9):e0222566.
6. Waiswa P. Understanding newborn care in Uganda: Towards future interventions. Karolinska Institutet (Sweden); 2010.
7. Kananura RM, Tetui M, Mutebi A, Bua JN, Waiswa P, Kiwanuka SN, Ekirapa-Kiracho E, Makumbi F. The neonatal mortality and its determinants in rural communities of Eastern Uganda. Reproductive health. 2016 Dec;13:1-9.
8. UNICEF. UNICEF Data Warehouse. Available from: https://data.unicef.org/resources/data_explorer/unicef_f/. Accessed 5 October 2021.
9. Zambia Statistics Agency ZSA, Ministry of Health MOH, University Teaching Hospital Virology Laboratory U-V, Icf. Zambia Demographic and Health Survey 2018. Lusaka, Zambia: ZSA, MOH, UTH-VL and ICF; 2020.
10. Kc A, Jha AK, Shrestha MP, Zhou H, Gurung A, Thapa J, Budhathoki SS. Trends for neonatal deaths in Nepal (2001–2016) to project progress towards the SDG target in 2030, and risk factor analyses to focus action. Maternal and child health journal. 2020 Feb;24:5-14.
11. Grady SC, Frake AN, Zhang Q, Bene M, Jordan DR, Vertalka J, Dossantos TC, Kadhim A, Namanya J, Pierre LM, Fan Y. Neonatal mortality in East Africa and West Africa: a geographic

analysis of district-level demographic and health survey data. Geospatial health. 2017 May 26;12(1).
12. Tembo D, Abobo FD, Kaonga P, Jacobs C, Bessing B. Risk factors associated with neonatal mortality among neonates admitted to neonatal intensive care unit of the University Teaching Hospital in Lusaka. Scientific Reports. 2024 Mar 4;14(1):1-2.
13. Kamanga A, Ngosa L, Aladesanmi O, Zulu M, McCarthy E, Choba K, Nyirenda J, Chizuni C, Mwiche A, Storey A, Shakwelele H. Reducing maternal and neonatal mortality through integrated and sustainability-focused programming in Zambia. PLOS Global Public Health. 2022 Dec 14;2(12):e0001162.
14. Guinsburg R, Sanudo A, Kiffer CR, Marinonio AS, Costa-Nobre DT, Areco KN, Kawakami MD, Miyoshi MH, Bandiera-Paiva P, Balda RD, Konstantyner T. Annual trend of neonatal mortality and its underlying causes: population-based study–São Paulo State, Brazil, 2004–2013. BMC pediatrics. 2021 Jan 26;21(1):54.
15. Norris M, Klabbers G, Pembe AB, Hanson C, Baker U, Aung K, Mmweteni M, Mfaume RS, Beňová L. A growing disadvantage of being born in an urban area? Analysing urban–rural disparities in neonatal mortality in 21 African countries with a focus on Tanzania. BMJ Global Health. 2022 Jan 1;7(1):e007544.
16. Shumba S, Moonga V, Miyoba TO, Jere S, Nkonde JM, Mumba P (2024) Socio-economic disparities and predictors of fertility among adolescents aged 15 to 19 in Zambia: Evidence from the Zambia Demographic and Health Survey (2018). PLOS Glob Public Health 4(3): e0002739. https://doi.org/10.1371/journal.pgph.0002739
17. Kujala S, Waiswa P, Kadobera D, Akuze J, Pariyo G, Hanson C. Trends and risk factors of stillbirths and neonatal deaths in Eastern Uganda (1982–2011): a cross-sectional, population-based study. Tropical Medicine & International Health. 2017 Jan;22(1):63-73.
18. Saloio CÁ, Morais Neto OL, Gonçalves DA, Bessa HE, Coelho Júnior JP, Afonso MS, Carvalho SR. Magnitude and determinants of neonatal and postneonatal mortality in Goiânia, Goiás, Brazil: a retrospective cohort study, 2012. Epidemiologia e Serviços de Saúde. 2020 Nov 16;29:e2020132.
19. Shumba S, Fwemba I, Kaymba V (2024) Spatial-temporal patterns and predictors of timing and inadequate antenatal care utilization in Zambia: A Generalized Linear Mixed Model (GLMM) investigation from 1992 to 2018. PLOS Glob Public Health 4(10): e0003213. https://doi.org/10.1371/journal.pgph.0003213
20. Miyoshi Y, Matsubara K, Takata N, Oka Y. Baby survival in Zambia: stillbirth and neonatal death in a local hospital setting. BMC pregnancy and childbirth. 2019 Dec;19:1-6.
21. Saleem S, Naqvi F, McClure EM, Nowak KJ, Tikmani SS, Garces AL, Hibberd PL, Moore JL, Nolen TL, Goudar SS, Kumar Y. Neonatal deaths in infants born weighing≥ 2500 g in low and middle-income countries. Reproductive Health. 2020 Nov;17:1-4.
22. Shumba S, Jacobs C, Kapembwa M, Miyoba TO, Musonda NC. Spatial analysis and associated factors of underweight, overweight and obesity among non-pregnant women of reproductive age in Zambia. African Health Sciences. 2024 Oct 6;24(3):292-301.
23. Shiferaw K, Mengistie B, Gobena T, Dheresa M, Seme A. Neonatal mortality rate and its Determinants: A community–based panel study in Ethiopia. Frontiers in Pediatrics. 2022 May 23;10:875652.
24. Mosley WH, Chen LC. An analytical framework for the study of child survival in developing countries. 1984. Bull World Health Organ. 2003;81(2):140-5. PMID: 12756980; PMCID: PMC2572391.

25. https://dhsprogram.com/topics/wealth-index/Wealth-Index-Construction.cfm.
26. Snijders TA, Bosker R. Multilevel analysis: An introduction to basic and advanced multilevel modeling.2011
27. UNICEF. (2023). *The State of the World's Children 2023: For Every Child, Health, Education, and Protection*.
28. WHO. (2022). *Maternal and Neonatal Health Guidelines*.
29. Shumba S, Moonga V, Miyoba TO, Jere S, Nkonde JM, Mumba P. Socio-economic disparities and predictors of fertility among adolescents aged 15 to 19 in Zambia: Evidence from the Zambia demographic and health survey (2018). PLOS Global Public Health. 2024 Mar 27;4(3):e0002739.
30. Kota K, Chomienne MH, Geneau R, Yaya S. Socio-economic and cultural factors associated with the utilization of maternal healthcare services in Togo: a cross-sectional study. Reproductive Health. 2023 Jul 25;20(1):109.
31. Smith, J. A., & Jones, R. L. (2020). The impact of maternal ART on neonatal outcomes: A systematic review. Journal of Maternal-Fetal & Neonatal Medicine.
32. Brown, T., & Green, D. (2019). Socioeconomic factors affecting neonatal mortality in HIV-positive mothers. Global Health Action.
33. Anderson, K. L., & Taylor, B. (2021). Access to healthcare and its impact on maternal and neonatal health in sub-Saharan Africa. *BMC Public Health*.
34. Ngwenya, N., & Makhubele, B. (2018). The role of socio-economic status in healthcare access and ART adherence among HIV-positive women. *AIDS Care*.
35. Chileshe, M., & Kasonde, M. (2022). Disparities in healthcare access for HIV-positive mothers in Zambia: Implications for neonatal health. *Zambia Medical Journal*.
36. Tende, M., & Mumba, C. (2020). The influence of socio-economic factors on health outcomes for HIV-positive mothers: A Zambian perspective. *African Journal of Health Sciences*.
37. Tembo D, Abobo FD, Kaonga P, Jacobs C, Bessing B. Risk factors associated with neonatal mortality among neonates admitted to neonatal intensive care unit of the University Teaching Hospital in Lusaka. Scientific Reports. 2024 Mar 4;14(1):5231.
38. Shumba S, Jacobs C, Kapembwa M, Miyoba TO, Musonda NC. Spatial analysis and associated factors of underweight, overweight and obesity among non-pregnant women of reproductive age in Zambia. African Health Sciences. 2024 Oct 6;24(3):292-301.
39. Hug L, Alexander M, You D, Alkema L. National, regional, and global levels and trends in neonatal mortality between 1990 and 2017, with scenario-based projections to 2030: a systematic analysis. The Lancet Global Health. 2019 Jun 1;7(6):e710-20.
40. UNICEF. UNICEF Data Warehouse. Available from: https://data.unicef.org/resources/data_explorer/unicef_f/. Accessed 5 October 2021.
41. WHO. Improving maternal and newborn health and survival and reducing stillbirth - Progress report 2023. https://www.who.int/publications/i/item/9789240073678
42. Bech CM, Stensgaard CN, Lund S, Holm-Hansen C, Brok JS, Nygaard U, Poulsen A. Risk factors for neonatal sepsis in Sub-Saharan Africa: a systematic review with meta-analysis. BMJ open. 2022 Sep 1;12(9):e054491.

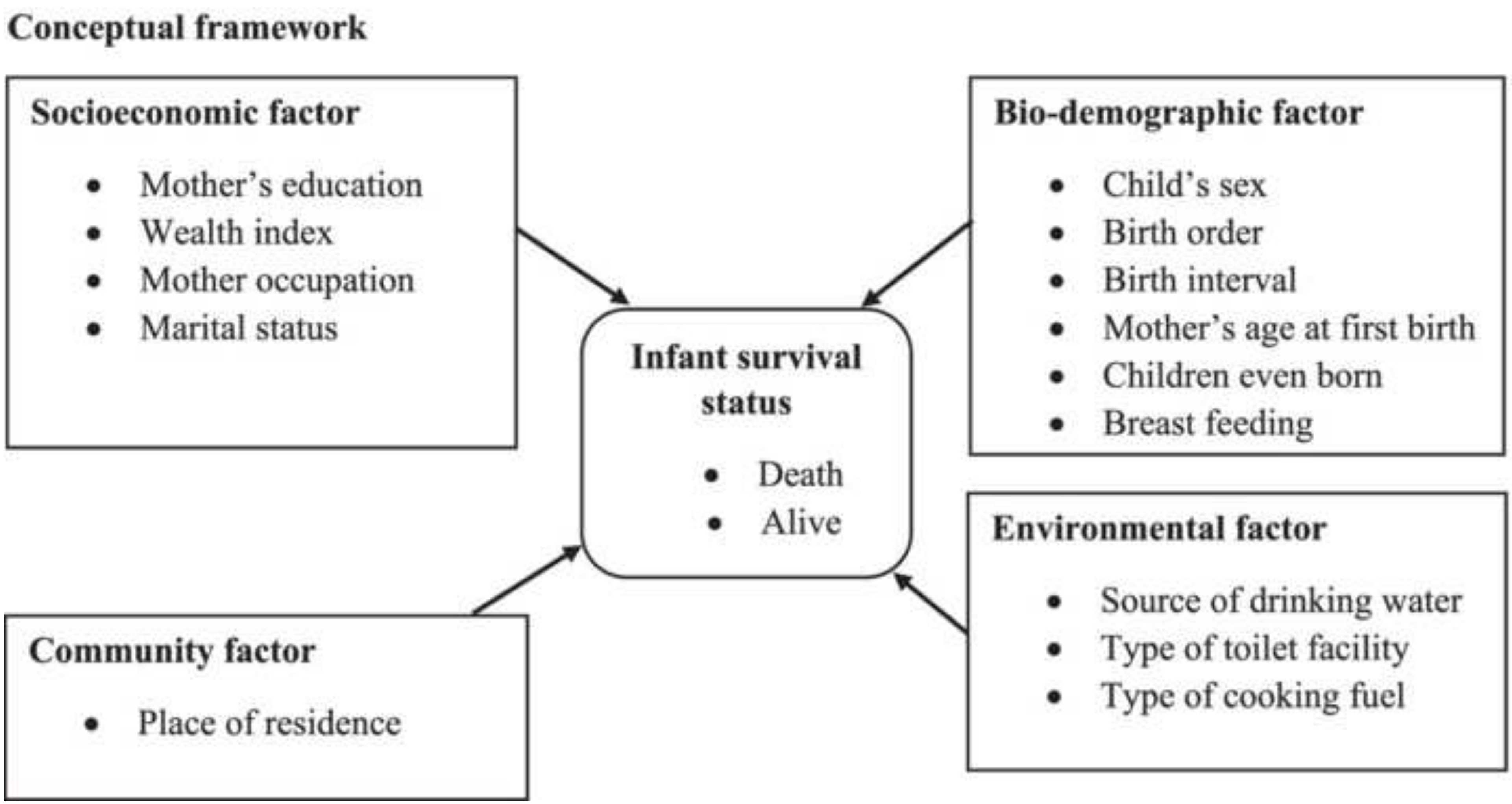
Conceptual framework
Socioeconomic factor
Mother's education
Wealth index
Mother occupation
Marital status
Bio-demographic factor
Child's sex
Birth order
Birth interval
Mother's age at first birth
Children even born
Breast feeding
Infant survival status
Death
Alive
Environmental factor
Source of drinking water
Type of toilet facility
Type of cooking fuel
Community factor
Place of residence

Description of sample derivation criteria

Click here to access/download;Figure;Fig 2.tiff

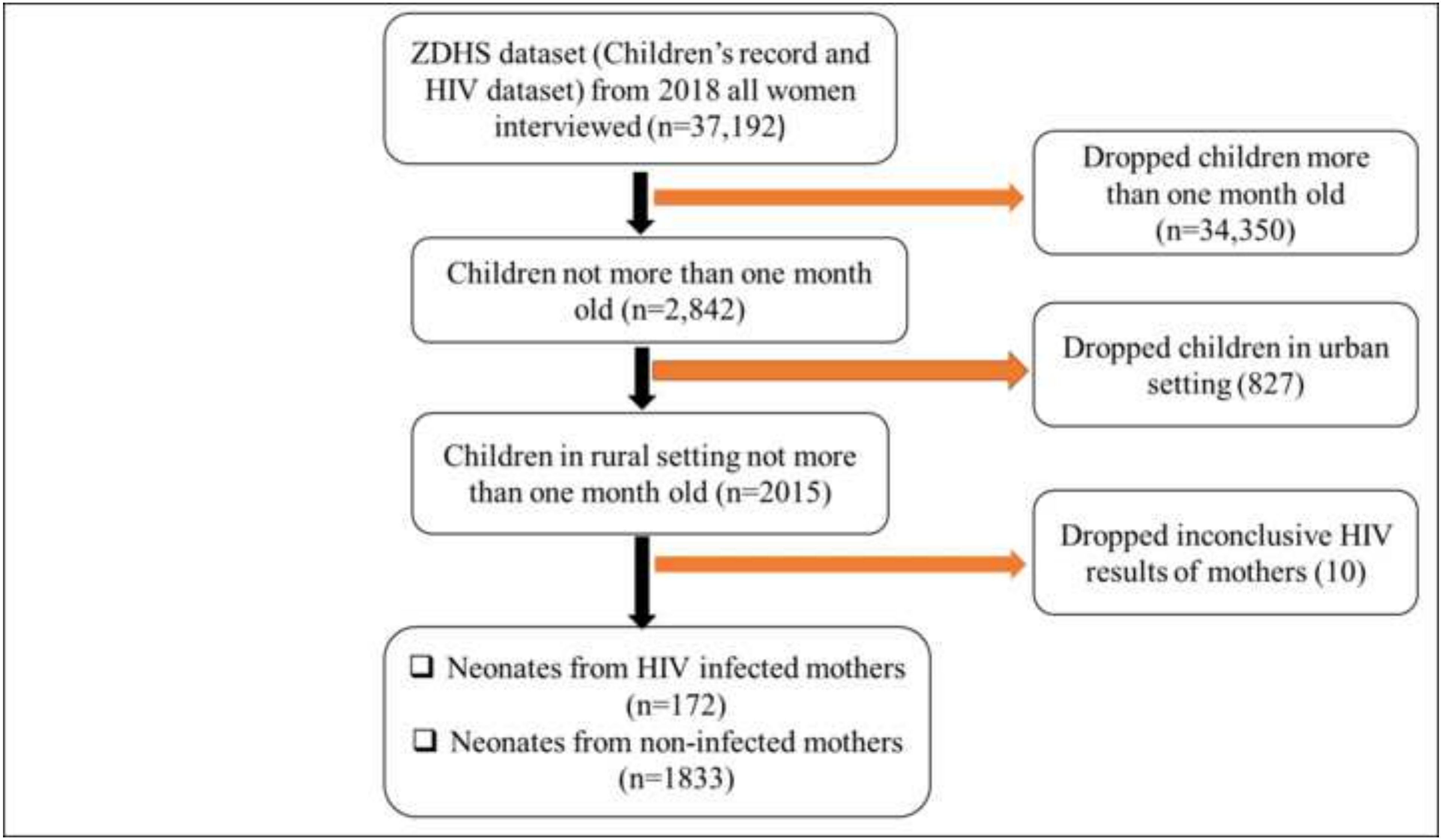

Proportion of neonatal mortality among HIV infected mothers in rural Zambia (2018)

Click here to access/download;Figure;Fig 3.tif

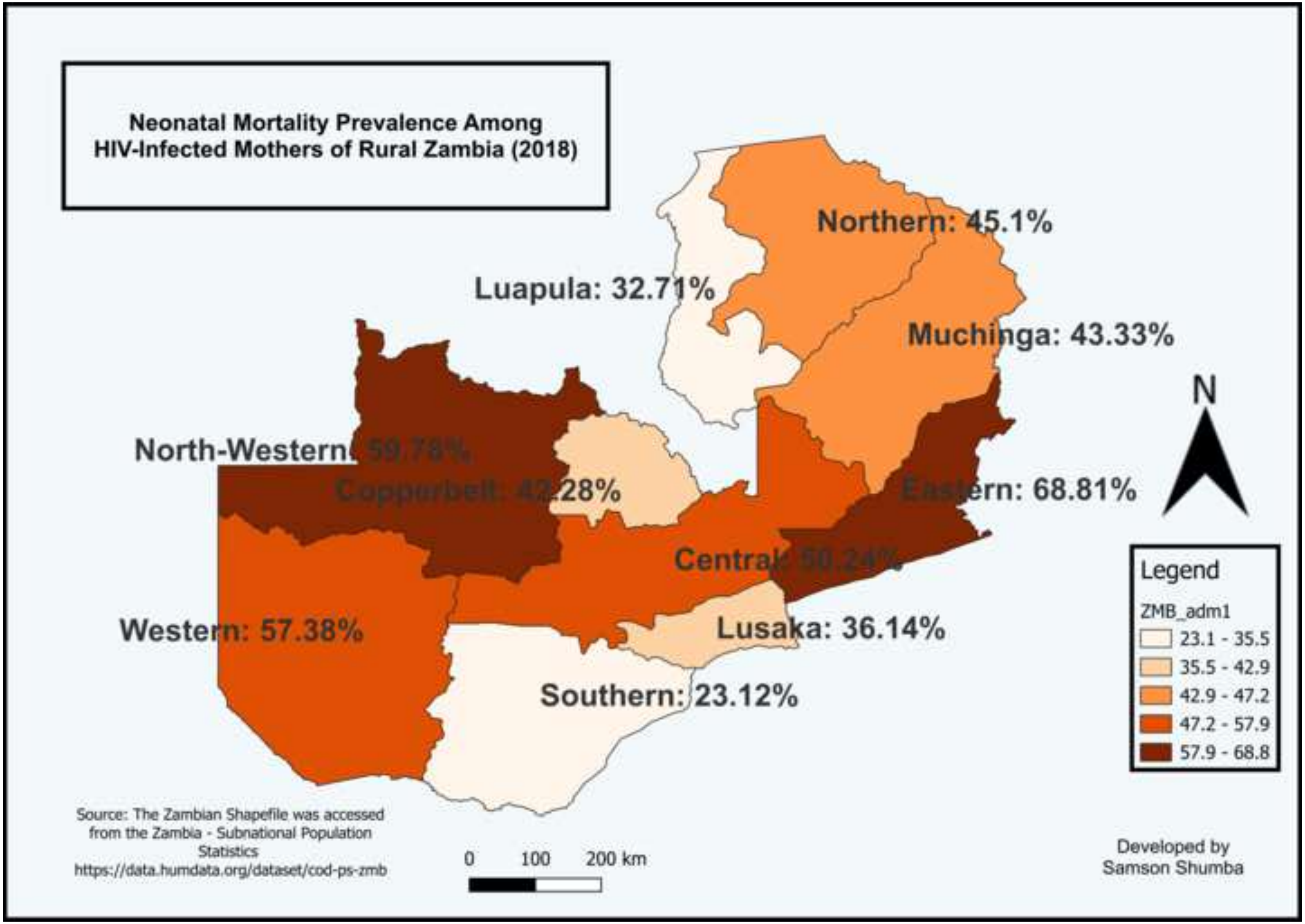

Proportion of neonatal mortality among non HIV-infected mothers in rural Zambia (2018)

Click here to access/download;Figure;Fig 4.tif

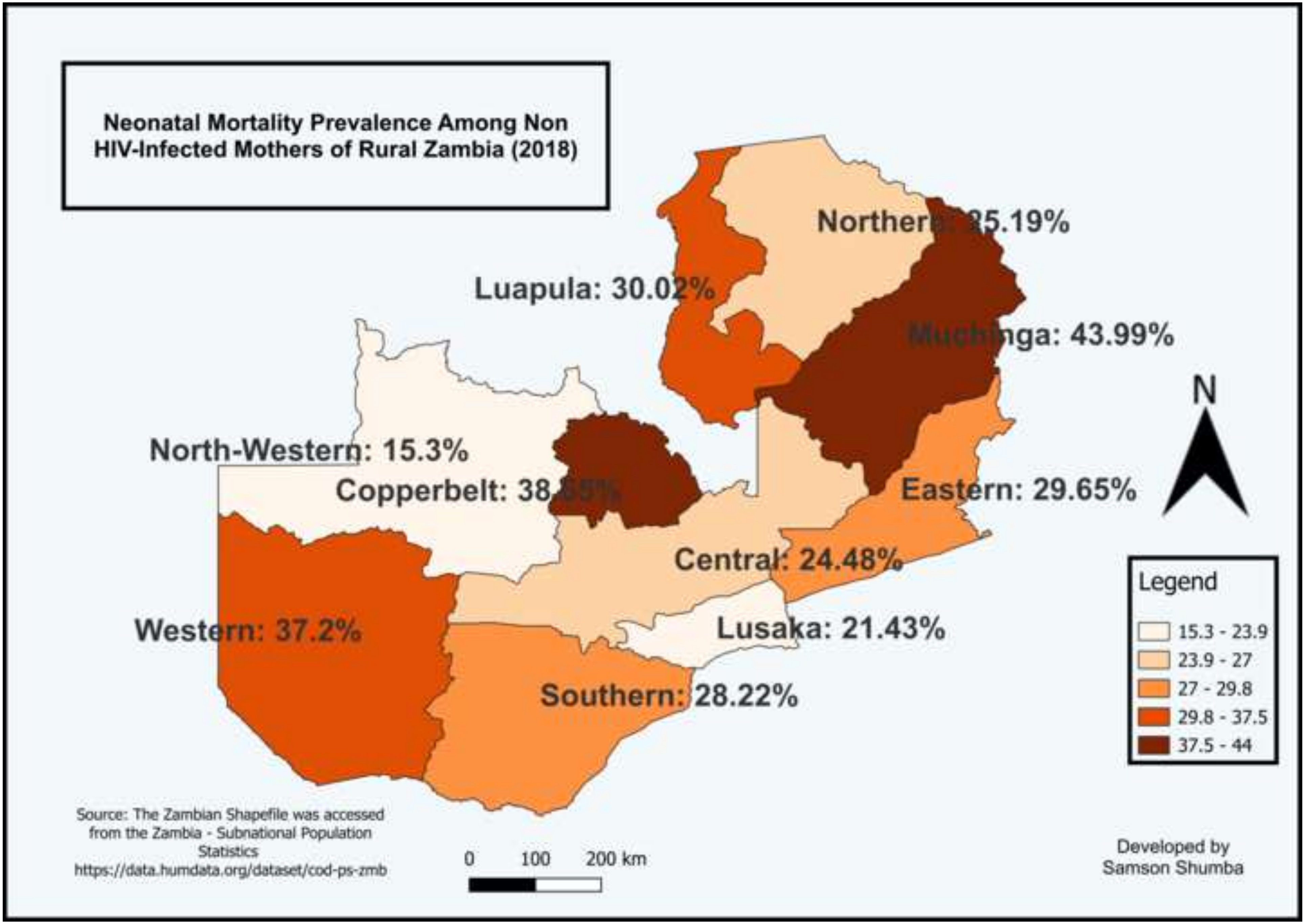

Click here to access/download
Supporting Information
AuthLetter_202305.pdf

Spatial Variations and Predictors of Neonatal Mortality among HIV-Infected and Non-Infected Mothers in Rural Zambia.Geospatial patterns and predictors of neonatal mortality among HIV infected and non-infected mothers of rural Zambia: A comparative analysis of the 2018 Zambia Demographic and Health Survey

Formatted: Font: (Default) Times New Roman, 18 pt, Bold

Formatted: Width: 8.5", Height: 11"

Formatted: Font: 18 pt

Formatted: Font: (Default) Times New Roman, 18 pt, Bold

Formatted: Font: 18 pt

Formatted: Centered

Samson Shumba[1], Deborah Tembo[2], Miyanda Simwaka[3], Nedah Chikonde Musonda[4], Chipo Nkwemu[5] and Sebean Mayimbo[6]

1. Department of Epidemiology & Biostatistics, School of Public Health, University of Zambia, Lusaka, Zambia.
2. Department of Emergency Preparedness and Response. Zambia National Public Health Institute, Lusaka, Zambia.
3. Ministry of Health, Lusaka, Zambia.
4. Department of Youth and Gender, Copperrose Zambia.
5. Centre for Infectious Disease Research in Zambia
6. Department of Midwifery, Women and Child Health, School of Nursing Sciences, University of Zambia, Lusaka, Zambia.

Corresponding Author

Samson Shumba

Samsonshumba1@gmail.com

Abstract

In Zambia, neonatal mortality still remains a noteworthy public health problem with a current rate of 27 deaths per 1000 , and ~~Neonatal mortality is the death of a live-born infant within the first 28 completed days of life. Neonatal mortality remains a major public health concern in most African countries, with the Sub Saharan region being the highest contributor at 27 deaths per 1000 live births, accounting for 43% of the total number of deaths. Zambia still fares poorly in terms of neonatal mortality,~~ ranking 162 out of 195 countries globally. The study aimed to investigate the spatial variation~~pattern~~s and predictors of neonatal mortality in rural Zambia among HIV-infected and non-infected mothers. This study utilized the national-level data from the 2018 Zambia Demographic and Health Survey (ZDHS)~~ program, utilizing the survey dataset from 2018~~. Statistical analys~~e~~es were conducted using the Rao – Scott Chi-square test to assess associations between ~~dependent~~ neonatal mortality and~~ independent~~ categorical variables. Additionally, a multilevel mixed effect logistic regression model was used to examine predictors of neonatal mortality. Geospatial variation~~pattern~~s of neonatal mortality across Zambia's ten provinces were mapped~~investigated~~ using Quantum Geographical Information System (QGIS) version 3.34.1~~ to generate a univariate choropleth map~~. Data analysis was performed using Stata version 14.2. The study findings indicate a higher mortality rate among HIV-infected mothers aged 45 to 49 (100%) compared to 95.21% among non-HIV-infected mothers. Neonatal mortality was notably elevated among neonates born to mothers with no education (40.78%) and those with HIV infection (54.56%). Employment status also influenced mortality rates, with employed non-HIV-infected mothers showing 36.35% mortality compared to 49.39% among unemployed HIV-infected mothers. Higher birth weights, particularly 4000 grams or higher, were associated with increased mortality for both HIV-infected (81.15%) and non-infected (21.00%) mothers. Multilevel logistic regression identified predictors of mortality, including maternal age (20-24~~40 to 44~~ years), neonate's sex (female),~~ and~~ duration of breastfeeding, maternal education and number of children

Formatted: Pattern: Clear (Background 1)

Formatted: Font: (Default) Times New Roman, Pattern: Clear (Background 1)

Formatted: Pattern: Clear (Background 1)

ever born~~maternal HIV status~~. Geospatial analysis revealed Eastern and North-Western provinces as hotspots for neonatal mortality among HIV-infected mothers, while Muchinga was a hotspot for non-HIV-infected mothers. This study examined neonatal mortality among HIV-infected and non-infected mothers in rural Zambia, uncovering predictors such as maternal age, neonate sex, birthweight, maternal education, duration of breastfeeding and number of children ever had~~HIV status, and community desired number of children~~. Regional variations in mortality underscore the necessity for targeted interventions. Strengthening healthcare infrastructure, community outreach, healthcare worker training, maternal education, and addressing regional differences are crucial for improving maternal and child health and meeting Sustainable Development Goals targets.

Formatted: Font: (Default) Times New Roman

Keyword: spatial, neonatal, mortality, HIV

Formatted: Left

Introduction

Neonatal mortality is characterized by the death of a live-born infant, irrespective of the gestational age at birth, within the initial 28 completed days of life [1]. The initial month after birth represents a critical phase for child survival, marked by the unfortunate loss of 2.4 million newborns globally in the year 2020 [2]. During that same year, nearly 47% of all deaths in children under the age of five occurred during the neonatal period, which spans the first 28 days of life and 98% of the deaths occurred in low and middle-income countries (LMIC) [1-3]. This percentage reflects a notable increase from the 40% reported in 1990. The world has made impressive progress towards the survival of children since 1990. In 2020, the number of neonatal deaths declined from 5 million in 1990 to 2.4 million, nonetheless, the decline in neonatal mortality from 1990 to 2020 has been slower than that of the post-neonatal under-five mortality [1].

Sub-Saharan Africa bears the highest global neonatal mortality rate, reaching approximately 27 deaths per 1000 live births, and plays a significant role in global newborn fatalities, accounting for 43% of the total [4,5]. In close proximity, central and southern Asia report a neonatal mortality rate of 23 deaths per 1000 live births, contributing to 36% of global newborn deaths [1]. Moreover, a child born in Sub-Saharan Africa faces a staggering tenfold higher likelihood of dying in the first month than a child born in a high-income country. In Uganda a study revealed that the underlying causes of death are related to poor access and low utilization of health services during pregnancy

and childbirth [6]. Furthermore, findings have revealed that more newborn deaths occur at home among the rural poor [7].

Zambia relative to other countries fairs poorly ranking 162 out of 195 countries globally in terms of neonatal mortality in Zambia [8]. According to Zambia Statistical Agency (2018) it indicated that neonatal mortality rate decreased from 37 to 27 neonatal deaths per 1000 live births between 2001 and 2018 [9]. Moreover, various studies have identified several factors correlated with heightened neonatal mortality rates in both the general population and facility-based settings. These include lower levels of maternal education [10-12], place of delivery and residence [13,14], maternal age [15,16], parity [17], gestational age [10,15], inadequate antenatal visits [18,19], newborn sex [13], newborn age [19], low birth weight [20], maternal and fetal complications [20], preterm birth [21], maternal BMI [22] and hypothermia [23]. Despite these findings, there remains a gap in understanding neonatal mortality and its associated risk factors, particularly within rural communities, especially between HIV-infected and non-infected mothers in rural Zambia. Therefore, this study aimed to investigate the geospatial patterns and predictors of neonatal mortality among both HIV-infected and non-infected mothers in rural Zambia.

~~Zambia relative to other countries fairs poorly ranking 162 out of 195 countries globally in terms of neonatal mortality in Zambia [8]. According to Zambia Statistical Agency (2018) it indicated that neonatal mortality rate decreased from 37 to 27 neonatal deaths per 1000 live births between 2001 and 2018 [9]. Moreover, various studies have identified several factors correlated with heightened neonatal mortality rates in both the general population and facility-based settings. These include lower levels of maternal education [10-12], place of delivery and residence [13,14], maternal age [15], parity [16], gestational age [10,15], inadequate antenatal visits [17], newborn sex [13], newborn age [18], low birth weight [19], maternal and fetal complications [18], preterm birth [19], and hypothermia [20]. Despite these findings, there remains a gap in understanding neonatal mortality and its associated risk factors, particularly within rural communities, especially between HIV-infected and non-infected mothers in rural Zambia. Therefore, this study aimed to investigate the geospatial patterns and predictors of neonatal mortality among both HIV-infected and non-infected mothers in rural Zambia.~~

Conceptual Framework

We modified the conceptual framework of neonatal mortality using the analytical framework established by Mosley and Chen. Originally proposed in 1984, this analytical framework for child mortality was subsequently adapted to emphasize variations in infant and child mortality attributable to socio-economic differentials, bio-demographic factors, household environmental conditions, nutrient deficiencies, and community influences (Fig 1) [24].

Formatted: Font: (Default) Times New Roman, 12 pt
Formatted: Space Before: Auto, After: Auto
Formatted: Font: (Default) Times New Roman, 12 pt
Formatted: Font: (Default) Times New Roman, 12 pt

Figure 2: Conceptual framework

Legend. Figure 1: Neonatal mortality conceptual framework

(Source: Mosley and Chen, 2003)

Formatted: Left, Space Before: Auto, After: Auto, Line spacing: single
Formatted: Font:

Methods

This study constitutes a secondary analysis of microdata utilizing national-level data sourced from the Zambia Demographic and Health Survey (ZDHS) program using the survey dataset in 2018. The ZDHS is a comprehensive, nationally representative household survey conducted by the Zambia Statistics Agency in collaboration with global partners, including ICF International and the United States Agency for International Development (USAID). The survey employs a two-stage sampling process, initially selecting enumeration areas (EAs) and subsequently households.

Dependent and independent variables

The primary outcome of interest in this study is neonatal mortality, defined as the death of infants within the first 28 days of life. The explanatory variables used are grouped into demographic, socio-economic, behavioral, and community-level factors to provide a holistic view of the determinants of neonatal mortality. Demographic variables include the mother's age (categorized as 15-19, 20-24, 25-29, 30-34, 35-39, 40-44, or 45-49 years), child's sex (male or female), and the number of children ever born (birth order). These variables capture biological and family composition factors that influence neonatal outcomes. Socio-economic factors encompass maternal education (none, primary, secondary/tertiary), household wealth index (poorest, poorer, middle, richer, richest), marital status (married or not married), and employment status (employed or unemployed). These indicators reflect the mother’s access to resources and social determinants

Formatted: Space Before: Auto, After: Auto

of health. Behavioral factors focus on health-related practices, including the duration of breastfeeding (ever vs. never breastfed). These behaviors are critical in understanding neonatal care practices that affect survival during the first month of life. Community-level factors consist of the source of drinking water (improved or unimproved), place of delivery (home, private, or public health facility) and region. These variables capture the environmental and infrastructural context in which the mother and newborn reside, which can significantly affect health outcomes [25]. The variable of interest in this study is neonatal mortality (that is death of babies between 0 and 28 days). The explanatory variables that were used in the study were demographic, socio-economic, behavioral, and community level factors. These variables included the following: the mother's age (categorized as 15-19, 20-24, 25-29, 30-34, 35-39, 40-44, or 45-49 years), current marital status of the mother (categorized as not married or married), the residence of the mother (categorized as urban or rural), education level of the mother (classified as no education, primary, secondary, tertiary), employment status of the mother (categorized as employed or unemployed), and household wealth index of the mother (categorized as poorest, poorer, middle, richer, or richest). The household wealth index is computed based on readily available data concerning a household's possession of selected assets, such as televisions and bicycles; materials utilized for housing construction; and types of water access and sanitation facilities [21].

Formatted: Space After: 0 pt

The selection of these variables bio-demographic, socio-economic, environmental, and community-level factors aligns with existing research on neonatal mortality and reflects key determinants recognized by global health organizations such as the World Health Organization (WHO) and UNICEF. Each variable captures essential dimensions that influence maternal and child health outcomes, which are critical to informing policies and interventions. **Community level variables**

Formatted: Font: (Default) Times New Roman, 12 pt

Formatted: Font: (Default) Times New Roman, 12 pt

Formatted: Font: (Default) Times New Roman, 12 pt

Formatted: Font: Not Bold

Community level variables in this study were derived by aggregating individual level data into clusters and incorporated community poverty, community education, community knowledge of family planning (FP) methods, and place of residence. These community-level variables were dichotomized as either 'low' or 'high,' reflecting the extent of the phenomena under investigation at the cluster level. Place of residence and geographical region were maintained in their original

Formatted: Space After: 0 pt

categorizations. Place of residence played a pivotal role in the sample design, as it was utilized as a criterion to estimate the prevalence of key demographic and health indicators at the national level. It was categorized as either 'rural' or 'urban' and directly contributed to the description of community characteristics.

Data Analysis

For descriptive analysis, frequencies and percentages were calculated for categorical variables to summarize the data. To assess the association between neonatal mortality (the outcome variable) and categorical predictors, the Rao–Scott Chi-square test was employed, accounting for the complex survey design. A multilevel mixed-effects logistic regression model was used to identify individual and community-level factors influencing neonatal mortality, recognizing the hierarchical structure of the data. The random factor in the study was cluster number coded as "v001".

The study followed an investigator-led approach for variable selection, drawing on a comprehensive review of relevant literature to ensure the inclusion of theoretically and empirically relevant predictors. Model selection was guided by the Akaike Information Criterion (AIC) and Bayesian Information Criterion (BIC), with the Intra-Class Correlation (ICC) used to quantify the extent of variance explained at the community level. These measures ensured the identification of the most appropriate and parsimonious model. All analyses were performed using Stata version 14.2.

Furthermore, spatial variations of neonatal mortality across provinces in Zambia was done using the Quantum Geographic Information System (QGIS) version 3.34.1. The coordinate system utilized was the World Geographic System (WGS) 1984 Universal Transverse Mercator (UTM) Zone 36S.

Model Specification for Multilevel Mixed Effect Logistic Regression

$$logit\left(P(Y_{ij}=1)\right)=\beta_0+\sum_{k=1}^{k}\beta_k X_{ijk}+u_j$$

Formatted: Justified, Line spacing: 1.5 lines
Formatted: Font: Not Bold
Formatted: Font: Not Bold
Formatted: Font: Not Bold
Formatted: Font: Not Bold
Formatted: Space Before: 0 pt
Formatted: Font: Not Bold
Formatted: Font: Not Bold
Formatted: Font: Not Bold
Formatted: Font: Not Bold
Formatted: Font: Not Bold
Formatted: Font: Not Bold
Formatted: Font: Not Bold
Formatted: Space Before: 10 pt
Formatted: Font: Bold
Formatted: Font: Bold
Formatted: Space Before: 0 pt

Where:

Logit is the log-odds function:

$$logit\left(P(Y_{ij}=1)\right)=\ln\left(\frac{P(Y_{ij}=1)}{1-P(Y_{ij}=1)}\right)$$

$u_j \sim N(0,\sigma_u^2)$ assumes that the cluster-level random effect follows a normal distribution with mean 0 and variance σ_u^2

Let:

- Y_{ij} = Outcome for individual i in cluster j (binary: Y_{ij} = 1 if the event occurs, and Y_{ij} = 0 otherwise).
- $X_{ij} = X_{ij1}, X_{ij2}, \ldots, X_{ijk}$ = vector of individual-level covariates
- β_0 = Fixed intercept (baseline log-odds of the outcome).
- β_k = Coefficients for individual-level predictors X_{ijk}
- u_j = Random intercept for cluster jjj, accounting for unobserved heterogeneity among clusters [26].

Intra-class Correlation ICC

The ICC quantifies the proportion of the total variance in the outcome attributable to differences between clusters:

$$ICC = \frac{\sigma_u^2}{\sigma_u^2+\frac{\pi^2}{3}}$$

Here $\frac{\pi^2}{3}$ is the variance of the logistic distribution at the individual level [23].~~**For descriptive purposes, frequencies and percentages were computed for categorical variables. To determine the association between the outcome variable (neonatal mortality) and the categorical variables, the Uncorrelated Design Based Chi-square test (Rao – Scott Chi-square test) was used. The multilevel mixed-effect logistic regression model was employed to determine the factors affecting newborn mortality. Additionally, the study used an investigator-led approach, all variables were selected from a wide range of literature. The Intra-Class Correlation (ICC), Akaike Information Criteria (AIC), and the Bayesian Information Criteria (BIC) were sufficiently explored to select the best-fit model. Stata version 14.2 was used for analysis.**~~

Formatted: Font: (Default) Times New Roman, 12 pt
Formatted: List Paragraph, Left, Space Before: 0 pt, Line spacing: single
Formatted: List Paragraph, Centered, Space Before: 0 pt, Line spacing: single
Formatted: Font: Times New Roman, Not Italic
Formatted: List Paragraph, Left, Space Before: 0 pt, Line spacing: single
Formatted: Left, Space Before: 0 pt, After: 8 pt, Line spacing: Multiple 1.08 li
Formatted ...
Formatted ...
Formatted: List Paragraph, Left, Space Before: 0 pt, Bulleted + Level: 1 + Aligned at: 0.25" + Indent at:
Formatted: Font: (Default) Times New Roman, 12 pt
Formatted ...
Formatted ...
Formatted: Font: (Default) Times New Roman, 12 pt
Formatted ...
Formatted ...
Formatted: List Paragraph, Line spacing: 1.5 lines, Bulleted + Level: 1 + Aligned at: 0.25" + Indent at:
Formatted: List Paragraph, Left, Space Before: 0 pt, Bulleted + Level: 1 + Aligned at: 0.25" + Indent at:
Formatted ...
Formatted ...
Formatted: List Paragraph, Left, Space Before: 0 pt, Line spacing: single, Bulleted + Level: 1 + Aligned at: 0.25" + Indent at: 0.5"
Formatted ...
Formatted: Left, Space After: 8 pt, Line spacing: Multiple 1.08 li
Formatted: Font: Not Bold
Formatted: Font: Not Bold
Formatted: List Paragraph, Left, Line spacing: single, Bulleted + Level: 1 + Aligned at: 0.25" + Indent at:
Formatted ...
Formatted: Font: (Default) Times New Roman
Formatted: List Paragraph, Left, Space Before: 0 pt, Line spacing: single

Geospatial Analysis

To examine the clustering and spatial distribution of neonatal mortality across provinces in Zambia, this study utilized Quantum Geographic Information System (QGIS) version 3.34.1 to construct a univariate choropleth map. Spatial analysis was conducted at the provincial level, with each neonate matched to their provincial residence using geo-coordinate data collected from the Demographic and Health Survey (DHS). The DHS employs pre-defined data assigning each case to a province. Spatial analysis units were defined as clusters of sample households as designated by the Zambian Demographic and Health Survey (ZDHS). The coordinate system utilized was the World Geographic System (WGS) 1984 Universal Transverse Mercator (UTM) Zone 36S.

Ethics statement

The methodologies used in the 2018 ZDHS, including biomarker measurement protocols, were ethically cleared by both the Inner City Fund (ICF) institutional review boards (IRBs) and the Tropical Diseases Research Centre (TDRC) in Zambia. Oral consent was obtained from respondents, with parental or guardian consent for adolescents under 18. Neonate information was collected from caregivers. Detailed information on the DHS consent process is available (https://www.dhsprogram.com/What-We-Do/Protecting-the-Privacy-of-DHS-Survey-Respondents.cfm). Authorization to use ZDHS data was granted by ICF Macro, and the dataset can be accessed (https://www.dhsprogram.com/data). The user strictly adhered to confidentiality guidelines, ensuring the anonymity of respondents.

Formatted: Normal (Web), Space Before: 0 pt, After: 13.5 pt, Pattern: Clear (White)

Results

Participation in the survey was limited to children aged one month from selected households of women who had consented to take part in the research. Detailed methods employed in the DHS are comprehensively documented elsewhere [9]. A total of 2015 participants were enrolled into the study comprising neonates from HIV-infected and non-infected mothers (as shown in Fig 2)1.

Figure 21: Description of sample derivation criteria

Legend. Figure 21: Description of sample derivation criteria

The map was generated using PowerPoint 2016. (Source: author generated). Dataset source: ZDHS women dataset accessible at: (https://www.dhsprogram.com/data).(Source: Author generated)

Formatted: Normal (Web), Left, Line spacing: single

Formatted: Pattern: Clear

The data presented in Table 12 provides an overview of neonatal mortality in rural settings among mothers infected with HIV and those not infected with HIV, drawing from the 2018 Zambia Demographic and Health Survey dataset. The findings reveal distinct patterns in neonatal mortality across various background characteristics, including maternal age, education level, employment status, neonate gender, birth order, birthweight, place of delivery, and HIV status. Notably, neonatal mortality increases significantly with maternal age ($p<0.0001$), with the highest mortality observed in the age group 45 to 49 (100%) among HIV-infected mothers compared to 95.21% in non-HIV infected mothers.

Furthermore, mortality rates were significantly elevated among non-HIV-infected mothers lacking formal education (40.78%), compared to 54.56% among HIV-infected mothers. Overall, neonatal mortality stood at 41.13% among mothers (both HIV and non-HIV-infected) with no formal education. Neonatal mortality was significantly higher among HIV-infected mothers who were not working (49.39%), in contrast to non-HIV-infected mothers, where high mortality was observed among those who were employed (36.36%).The neonatal mortality rate was high at 37.17% among neonates whose mothers (both HIV infected and non-infected mothers) were reported to be working. Neonatal mortality was also higher among males in both HIV-infected and non-infected mothers (52.99% and 33.24%, respectively). Moreover, neonatal mortality was consistently higher among children with a birth order of 7 or higher among HIV-infected and non-infected mothers in rural Zambia (81.15% and 61.82%), with overall mortality also reported high among those of the 7th or higher birth order (63.73%).

Additionally, neonatal mortality was reported higher among neonates born with a weight of 4000 grams or higher among HIV-infected and non-infected mothers (81.15% and 21.00%, respectively). Overall, neonatal mortality was higher among mothers infected with HIV (51.48%) compared to those not infected (29.89%).

~~**Table 2: Key attributes of neonatal mortality in rural setting of Zambia in 2018**~~**Table 1: Key attributes of neonatal mortality in rural setting of Zambia in 2018**

Variables	HIV – Infected (n=172)		Non-HIV infected (n=1,833)		Overall (n=2,005)	
	Alive	Dead	Alive	Dead	Alive	Dead
**Age of mother**	<0.0001*		0.0001*		<0.0001*	
15 - 19	2 (100)	0 (0.00)	247 (94.28)	15 (5.72)	249 (94.54)	15 (94.54)
20 - 24	23 (70.71)	9 (29.29)	341 (83.58)	67 (16.42)	372 (82.73)	78 (17.27)
25 – 29	11 (59.26)	8 (40.74)	230 (77.87)	65 (22.13)	248 (76.61)	76 (23.39)
30 – 34	22 (70.25)	9 (29.75)	182 (69.47)	79 (45.30)	214 (70.11)	91 (29.89)
35 – 39	18 (59.28)	12 (40.72)	117 (54.70)	97 (45.30)	136 (54.73)	113 (45.27)
40 – 44	8 (26.76)	21 (73.24)	60 (38.01)	98 (61.99)	68 (35.63)	122 (64.37)
45 - 49	0 (0.00)	18 (100.00)	4 (4.79)	81 (95.21)	4 (3.94)	99 (96.06)
**Education level of the mother**	0.584		<0.0001*		<0.0001*	
No Education	12 (45.44)	14 (54.56)	127 (59.22)	88 (40.78)	148 (58.87)	103 (41.13)
Primary	44 (48.62)	46 (51.38)	723 (68.56)	332 (31.44)	786 (67.10)	386 (32.90)
Secondary	26 (61.51)	16 (38.49)	317 (79.79)	80 (20.21)	351 (77.80)	100 (22.20)
Higher	1 (50.64)	1 (49.36)	13 (77.54)	4 (22.46)	15 (75.30)	5 (24.70)
**Marital status**	0.6842		0.1775		0.3384	
Not married	32 (54.13)	27 (45.87)	279 (73.61)	100 (26.39)	319 (70.94)	131 (29.06)
Married	51 (49.94)	50 (50.07)	902 (69.09)	404 (30.91)	981 (67.93)	463 (32.07)
**Wealth Index of the mother**	0.92		0.0821		0.1169	
Poorest	25 (51.17)	24 (48.83)	456 (68.68)	208 (31.32)	501 (67.91)	237 (32.09)
Poorer	27 (56.59)	21 (43.41)	407 (75.12)	135 (24.88)	440 (73.27)	160 (26.73)
Middle	21 (50.44)	20 (49.56)	224 (64.91)	121 (35.09)	256 (63.89)	145 (36.11)
Richer	7 (44.18)	8 (55.82)	66 (65.34)	35 (34.66)	73 (62.62)	43 (37.38)
Richest	3 (41.28)	5 (58.72)	27 (86.17)	4 (13.83)	32 (77.92)	9 (22.08)

Formatted Table

Formatted Table

Formatted Table

**Mother currently working**	0.8562		0.0001*		0.0002*	
No	41 (50.61)	40 (49.39)	678 (75.71)	218 (24.29)	739 (73.86)	261 (26.14)
Yes	41 (52.37)	38 (47.63)	503 (63.74)	286 (36.26)	562 (62.83)	333 (37.17)
**Sex of child**	0.1504		0.0048*		0.0003*	
Male	45 (47.01)	51 (52.99)	599 (66.76)	298 (33.24)	658 (64.77)	358 (35.23)
Female	37 (58.24)	27 (41.76)	582 (73.93)	205 (26.07)	642 (73.13)	236 (26.87)
**Birth Order**	0.0004*		<0.0001		<0.0001*	-
1-3rd birth order	37 (63.39)	21 (36.61)	682 (86.41)	107 (13.59)	741 (85.02)	130 (14.98)
4-6th birth order	39 (61.11)	25 (38.89)	338 (71.39)	136 (28.61)	390 (70.17)	166 (29.83)
7th + order	7 (18.85)	32 (81.15)	161 (38.18)	261 (61.82)	169 (36.27)	298 (63.73)
**Place of Delivery**	0.3923		0.015*		0.0187*	
Home	7 (81.33)	1 (18.67)	164 (83.53)	32 (16.47)	179 (83.87)	34 (16.13)
Private health facility	8 (100.00)	0 (0.00)	84 (95.45)	4 (4.55)	1003 (89.13)	122 (10.87)
Government	65 (79.26)	17 (20.74)	912 (89.91)	102 (10.09)	92 (95.83)	4 (4.17)
Unspecified	4 (100.00)	0 (0.00)	21 (82.61)	4 (17.39)	27 (81.92)	36 (18.08)
**Birth weight**	0.0004*		<0.0001*		<0.0001*	
Less than 2500	37 (63.39)	21 (36.61)	72 (80.83)	17 (19.17)	82 (80.71)	20 (19.29)
2500 - 3999	39 (61.11)	25 (38.89)	789 95.05)	41 (4.95)	862 (94.27)	52 (5.73)
4000+	7 (18.85)	32 (81.15)	319 (79.00)	85 (21.00)	357 (79.02)	95 (20.98)
**ANC visits**	0.7065		0.6983		0.8741	
<8 ANC visits	81 (86.97)	12 (13.02)	1142 (95.19)	58 (4.81)	1257 (94.59)	72 (5.41)
8+ ANC visits	1 (100.00)	0 (0.00)	17 (92.95)	1 (7.05)	19 (93.68)	1 (6.32)
**HIV Results**	-	-	-	-	<0.0001*	
HIV negative	NA	NA	NA	NA	1180.75	503 (29.89)
HIV positive	NA	NA	NA	NA	83 (51.48)	78 (48.52)
**Duration of breastfeeding**	<0.0001		<0.0001		<0.0001	
Ever breastfed	4 (25.03)	13 (74.97)	45 (38.37)	72 (61.63)	49 (36.66)	85 (63.34)
Never breastfed	1 (9.32)	6 (90.68)	5 (6.82)	71 (93.18)	6 (7.01)	77 (92.99)
Still breastfeeding	78 (100.00)	0 (0.00)	1131 (100.00)	0 (0.00)	1209 (100.00)	0 (0.00)
**Children ever born**	0.008		<0.0001		<0.0001	-

Formatted Table

Formatted Table

Formatted Table

Formatted: Font: (Default) Times New Roman

Formatted: Font: (Default) Times New Roman

Formatted: Font: (Default) Times New Roman

Formatted: Font: (Default) Times New Roman

Formatted Table

Formatted: Font: (Default) Times New Roman

Formatted: Font: (Default) Times New Roman

Zero to two	24 (68.46)	11 (31.54)	530 (89.77)	60 (10.23)	71 (11.41)	554 (88.59)
three to four	26 (57.09)	20 (42.91)	279 (77.62)	80 (22.38)	100 (24.71)	305 (75.29)
five to six	25 (62.64)	15 (37.36)	211 (67.38)	102 (32.62)	117 (33.16)	236 (66.84)
seven plus	7 (18.85)	32 (81.15)	161 (38.18)	261 (61.82)	293 (63.49)	168 (36.51)
**Source of drinking water**	0.4797	-	0.4745	-	0.696	-
Improved	61 (53.13)	54 (46.87)	625 (71.06)	255 (29.94)	309 (31.02)	686 (68.98)
Unimproved	21 (47.23)	24 (52.77)	556 (69.07)	249 (30.93	273 (32.09)	577 (67.91)
**Type of toilet facility**	0.0727	-	0.6089	-	0.3667	-
Improved	40(60.79)	25 (39.21)	428 (71.01)	175 (28.99)	467 (70.02)	200 (29.98)
Unimproved	43 (45.22)	53 (54.78)	753 (69.61)	329 (30.39)	796 (67.62)	381 (32.38)
**Type of cooking fuel**	0.0125	-	0.029	-	0.0056	-
safe	4 (100.00)	0 (0.00)	21 (88.17)	3 (11.83)	25 (88.63)	3 (11.37)
unsafe	79 (50.34)	78 (49.66)	1160 (69.85)	501 (30.15)	1239 (68.18)	578 (31.82)

All tests used the Rao – Scott Chi-square test, ANC: Antenatal Care, NA: Not Applicable

Formatted Table

Formatted: Font: (Default) Times New Roman

Formatted: Normal, Left, Line spacing: single, Pattern: Clear

Formatted: Font: (Default) Times New Roman

Geospatial analysis of neonatal mortality across rural Zambia among HIV-infected mothers

Formatted: Normal, Left, Line spacing: single, Pattern: Clear

The study examined the geospatial patterns of neonatal mortality among mothers infected with HIV in rural Zambia. It revealed that North-western and Eastern provinces exhibited a high proportion of neonatal mortality, followed by Western and Central provinces. In contrast, Southern and Luapula provinces reported the lowest rates of neonatal mortality among HIV-infected mothers (~~see~~ Fig 3~~2~~).

Legend. Figure 3~~2~~: Shows the proportion of neonatal mortality among HIV infected mothers in rural Zambia (2018)

The map was generated using QGIS version 3.34.1. (Source: author generated)

Geospatial analysis of neonatal mortality across rural Zambia among non-HIV infected mothers (2018)

The study revealed geospatial patterns of neonatal mortality among non-HIV-infected mothers in rural Zambia. Specifically, Muchinga provinces emerged as a frontrunner in terms of neonatal mortality rates, with Western, Copperbelt and Lusaka provinces following closely behind. Conversely, North-western, Luapula and Central provinces exhibited the lowest rates of neonatal mortality as shown in Fig 4.

Legend. Figure 4~~3~~: Shows the proportion of neonatal mortality among non HIV-infected mothers in rural Zambia (2018)

The map was generated using QGIS version 3.34.1. (Source: author generated)

Formatted: Left, Line spacing: single, Pattern: Clear

~~**Geospatial analysis of neonatal mortality across rural Zambia among HIV infected and non-HIV infected mothers**~~

~~The study conducted a spatial analysis of neonatal mortality among both HIV-infected and non-infected individuals. The results indicate that neonatal mortality rates were particularly high in~~

Muchinga and Western provinces, with Copperbelt and Eastern provinces following closely behind. Conversely, Lusaka and North Western provinces reported the lowest proportions of neonatal mortality (see Fig 4).

Legend. Figure 4: Shows the proportion of neonatal mortality among HIV infected and non-HIV infected mothers in rural Zambia (2018)

The map was generated using QGIS version 3.34.1. (Source: author generated)

Formatted: Left, Line spacing: single, Pattern: Clear

Multilevel mixed effect logistic regression of neonatal mortality amongof HIV-infected and non-infected mothers in rural Zambia

The adjusted model reveals several significant factors associated with neonatal mortality, controlling for other variables. Female neonates had a 65% lower chance of dying compared to males (AOR: 0.35, 95% CI: 0.15–0.84, p = 0.019), holding other factors constant. Births in public health facilities posed a significantly higher risk compared to home deliveries (AOR: 6.93, 95% CI: 1.58–30.45, p = 0.010). Similarly, neonates born to mothers with primary education had 88% lower odds of mortality than those whose mothers had no formal education (AOR: 0.12, 95% CI: 0.02–0.79, p = 0.028). Neonates who were never breastfed faced much higher mortality risks than those who were breastfed (AOR: 6.15, 95% CI: 1.89–19.98, p = 0.003). Regional variations were also apparent, as mothers from North Western Province had a significantly lower likelihood of neonatal mortality compared to those from Central Province (AOR: 0.14, 95% CI: 0.02–0.94, p = 0.043), indicating potential disparities in healthcare access or quality across regions.

Neonates born to HIV-positive mothers have almost five times higher odds of death compared to those born to HIV-negative mothers (AOR: 4.83, 95% CI: 0.90–25.91, p = 0.066), however we could not rule out chance finding. While this suggests that neonates born to HIV-positive mothers are nearly five times more likely to die, the wide confidence interval and p-value above 0.05 indicate that this result may lack precision and certainty. Other variables, such as maternal age, employment, and birthweight, did not show statistically significant associations with neonatal

mortality when holding other factors constant. For example, mothers aged 35–39 exhibited lower odds of neonatal mortality (AOR: 0.12, 95% CI: 0.01–1.14, p = 0.065) than younger mothers aged 15–19, but the p-value did not meet the 0.05 threshold for significance. Similarly, neonates born to HIV-positive mothers had higher odds of mortality (AOR: 4.83, 95% CI: 0.90–25.91, p = 0.066).

The findings from the study revealed an intra-class correlation coefficient (ICC) of 3.81×10^{-33} and an intercept of 1.25×10^{-32} for the random effect associated with cluster number. These values suggest that almost all the variability in neonatal mortality is not attributable to differences between clusters (enumeration areas) but rather to individual-level factors. In practical terms, this indicates a minimal clustering effect, meaning that the neonatal mortality rates across clusters are relatively homogeneous, and most of the differences arise from individual characteristics (e.g., maternal education, breastfeeding, or HIV status). Additionally, multicolinearity diagnostics were performed, with all variables in the adjusted model showing Variance Inflation Factors (VIFs) below 5, confirming that multicolinearity was not a concern. This ensures the reliability of the model's estimates by indicating that the independent variables are not excessively correlated with one another.

The study employed a multilevel logistic regression model to examine various factors associated with neonatal mortality. Results indicate that mothers aged 40 to 44 years had significantly higher odds of neonatal mortality compared to those aged 15 to 19 years (Adjusted Odds Ratio [AOR]: 3.12, 95% Confidence Interval [CI]: 1.43 – 164.14; p<0.05). Additionally, female neonates exhibited lower odds of mortality compared to males (AOR: 0.58; 95% CI: 0.34 – 0.98; p<0.05). Moreover, neonates with normal birth weight (2500g to 3999g) demonstrated decreased odds of mortality compared to those with low birth weight (less than 2500g) (AOR: 0.2; 95% CI: 0.08 – 0.50; p<0.05). Conversely, neonates born to mothers infected with HIV showed increased odds of mortality compared to those born to HIV-negative mothers (AOR: 2.42, 95% CI: 1.03 – 5.70; p<0.05). Furthermore, the study investigated community level factors and found that neonates from communities with a higher desired number of children had reduced odds of mortality compared to those from communities with a lower desired number of children holding all factors constant.

~~The study delved further into examining margin probabilities, revealing that the likelihood of neonatal mortality among HIV-infected mothers in rural Zambia was notably higher compared to non-infected mothers, with probabilities of 0.1 (95% CI, 0.04 – 0.16) and 0.05 (95% CI, 0.04 – 0.06), respectively. The selection of the best-fit model was determined through Akaike Information Criteria (AIC), where Model 3 (Individual plus community level model) was favored over Model 2 (individual level model) and Model 1 (the null model). Additionally, an Intraclass correlation (ICC) of 0.03 was identified, suggesting that merely 3% of the total variance in the outcome variable can be attributed to differences between groups, while the vast majority, 97%, stems from differences within groups. This indicates a low level of clustering or similarity within groups, signifying diverse outcomes within clusters with minimal resemblance among them.~~

Table 2~~3~~: Multilevel mixed effect logistic regression of neonatal mortality of HIV-infected and non-infected mothers in rural Zambia

Variables	COR (95% CI)	P-value	AOR (95% CI)	P-value
**Mother's age**	-	-	-	-
15 - 19	Ref (1)		Ref (1)	-
20 - 24	2.90 (1.70 - 4.94)	<0.001	3.49 (0.86 - 14.22)	0.081
25 -29	4.71 (2.75 - 8.07)	<0.001	1.63 (0.30 - 8.80)	0.570
30 - 34	7.34 (4.31 - 12.49)	<0.001	2.02 (0.32 - 12.70)	0.452
35 - 39	10.69 (6.21 - 18.41)	<0.001	0.12 (0.01 - 1.14)	0.065
40 -44	29.23 (16.51 - 51.77)	<0.001	3.88 (0.29 - 52.72)	0.309
45 - 49	399.36 (140.72 - 1133.39)	<0.001	-	-
**Wealth Index**				-
Poorest	Ref (1)		Ref (1)	-
Poorer	0.81 (0.62 - 1.05)	0.105	2.48 (0.80 - 7.68)	0.115
Middle	1.16 (0.87 - 1.55)	0.323	2.78 (0.77 - 10.04)	0.118
Richer	0.99 (0.61- 1.59)	0.959	9.31 (0.78 - 111.08)	0.078
Richest	0.72 (0.34 - 1.50)	0.386	8.19 (0.35- 189.69)	0.189
**Sex of child**				-
Male	Ref (1)		Ref (1)	-
Female	0.70 (0.57 - 0.87)	0.001	0.35 (0.15 - 0.84)	0.019*
**Place of Delivery**				-
Home	Ref (1)		Ref (1)	-
Private	0.19 (0.06 - 0.56)	0.003	0.50 (0.02 - 11.73)	0.667
Public	0.54 (0.36 - 0.82)	0.003	6.93 (1.58 - 30.45)	0.010*
**Education**				-
None	Ref (1)		Ref (1)	-
Primary	0.62 (0.46 - 0.84)	0.002	0.12 (0.02 - 0.79)	0.028*
Secondary/tertiary	0.33 (0.23 - 0.47)	<0.001	0.20 (0.02 - 1.72)	0.143

**Marital status**				-
Not married	Ref (1)		Ref (1)	-
Married	1.27 (0.98 - 1.63)	0.068	2.39 (0.79 - 7.19)	0.122
**Employment**				-
No	Ref (1)		Ref (1)	-
Yes	1.90 (1.53 - 2.37)	<0.001	1.57 (0.63 - 3.92)	0.335
**Birth order**				-
0 - 3	Ref (1)		Ref (1)	-
4 - 6	2.91 (2.22 - 3.80)	<0.001		-
7 +	10.66 (0.02 - 14.16)	<0.001	1.10 (0.14 - 8.69)	0.926
**Birthweight**				-
<2500	Ref (1)		Ref (1)	-
2500 - 3999	0.20 (0.11 - 0.36)	<0.001	0.42 (0.09 - 1.90)	0.258
4000 - 9999	1.19 (0.68 - 2.07)	0.55	2.38 (0.40 - 14.22)	0.343
**HIV status**				-
Negative	Ref (1)		Ref (1)	-
Positive	2.42 (1.69 - 3.47)	<0.001	4.83 (0.90 - 25.91)	0.066
**Toilet**				-
Improved	Ref (1)		Ref (1)	-
Unimproved	1.09 (0.86 - 1.39)	0.466	1.00 (0.34 - 2.96)	0.999
**Duration of breastfeeding**				-
Ever breastfed,	Ref (1)		Ref (1)	-
Never breastfed	6.08 (2.69 - 13.74)	<0.001	6.15 (1.89 - 19.98)	0.003*
**Source of drinking water**				-
Improved	Ref (1)		Ref (1)	-
Unimproved	1.00 (0.80 - 1.26)	0.967	0.95 (0.37 - 2.44)	0.909
**Region**				-
Central	Ref (1)		Ref (1)	-
Copperbelt	1.48 (0.77 - 2.85)	0.242	2.78 (0.15 - 51.91)	0.493
Eastern	1.32 (0.79 - 2.22)	0.294	1.63 (0.23 - 11.59)	0.623
Luapula	1.28 (0.74 - 2.19)	0.378	1.92 (0.27 - 13.80)	0.519
Lusaka	0.97 (0.48 - 1.95)	0.923	0.06 (0.003 - 1.26)	0.071
Muchinga	2.23 (1.30 - 3.82)	0.004	2.67 (0.46 - 15.68)	0.272
Northern	0.79 (0.45 - 1.40)	0.422	1.65 (0.22 - 12.48)	0.630
North Western	0.63 (0.34 - 1.19)	0.155	0.14 (0.02 - 0.94)	0.043*
Southern	0.99 (0.56 - 1.73)	0.965	2.10 (0.31 - 14.39)	0.449
Western	1.85 (1.08 - 3.17)	0.026	0.43 (0.08 - 2.41)	0.335

COR: Crude Odds Ratio AOR: Adjusted Odds Ratio

Formatted: Font: 10 pt, Not Bold

Field Code Changed

Variables	Model 1 (Null Model) OR	Model 2 (Competing model) AOR	Model 3 (Accepted Model) AOR
**Age of mother**	-	-	-
15–19	–	Ref (1)	Ref

20 – 24	–	1.53 (0.64 - 3.61)	1.59 (0.68 - 3.71)
25 – 29	–	1.53 (0.53 - 4.39)	1.57 (0.56 - 4.41)
30 – 34	–	1.75 (0.51 - 6.05)	1.93 (0.57 - 6.46)
35 – 39	–	1.06 (0.23 - 4.86)	1.11 (0.25 - 4.95)
40 – 44	–	2.99 (0.63 - 14.30)	3.12 (1.43 - 164.14)***
45 - 49	–	18.03 (1.61 - 202.03)	15.34 (1.43 - 164.14)
**Education level of the mother**	-	-	-
No Education	–	Ref (1)	Ref (1)
Primary	–	1.61 (0.67 - 3.93)	1.44 (0.62 - 3.36)
Secondary	–	1.95 (0.66 - 5.73)	1.58 (0.53 - 4.66)
Higher	–	5.64 (0.51 - 62.88)	3.50 (0.32 - 37.73)
Marital status			-
Not married		Ref (1)	Ref (1)
Married	–	0.94 (0.48 - 1.82)	0.92 (0.48 - 1.75)
**Wealth Index of the mother**	-	-	-
Poorest	–	Ref (1)	Ref (1)
Poorer	–	0.70 (0.35 - 1.38)	0.70 (0.36 - 1.35)
Middle	–	1.66 (0.79 - 3.49)	1.38 (0.63 - 3.02)
Richer	–	0.92 (0.20 - 4.24)	0.77 (0.17 - 3.55)
Richest	–	1.83 (0.15 - 22.91)	1.23 (0.11 - 14.17)
**Mother currently working**	-	-	-
No	–	Ref (1)	Ref (1)
Yes	–	0.97 (0.55 - 1.82)	1.16 (0.63 - 2.15)
**Frequency of listening to rad**	-	-	-
Not at all	–	Ref (1)	Ref (1)
<once a week	–	1.06 (0.43 - 2.59)	1.15 (0.48 - 2.75)
≥ once a week	–	1.22 (0.48 - 3.09)	1.21 (0.49 - 3.03)
Almost everyday	–	1.61 (0.71 - 3.63)	1.62 (0.74 - 3.56)
**Frequency of reading NS/MZ**	-	-	-
Not at all	–	Ref (1)	Ref (1)
<once a week	–	0.84 (0.26 - 2.73)	1.00 (0.32 - 3.11)
≥ once a week	–	1.04 (0.25 - 4.23)	0.89 (0.21 - 3.74)
Almost everyday	–	0.73 (0.05 - 9.45)	0.54 (0.04 - 6.94)
**Frequency of watching television**	-	-	-
Not at all	–	Ref (1)	Ref (1)
<once a week	–	0.41 (1.56 - 1.08)	0.53 (0.14 - 2.05)
≥ once a week	–	0.45 (0.14 - 1.41)	0.24 (0.03 - 1.93)
Almost everyday	–	0.31 (0.09 - 1.11)	0.46 (0.08 - 2.57)
**Sex of child**	-	-	-
Male	–	Ref (1)	Ref (1)

Female	–	0.55 (0.32 - 0.95)***	0.58 (0.34 - 0.98)***
**Birth Order**	-	-	-
1-3rd birth order	–	Ref (1)	Ref (1)
4-6th birth order	–	0.81 (0.44 - 1.51)	0.66 (0.26 - 1.62)
7th + order	–	2.39 (1.02 - 5.58)	1.47 (0.43 - 5.01)
**Place of Delivery**	-	-	-
Home	–	Ref (1)	Ref (1)
Government health facility	–	0.53 (0.10 - 2.75)	1.33 (0.64 - 2.76)
Private health facility	–	1.54 (0.72 - 3.28)	0.47 (0.09 - 2.51)
Unspecified	–	1.23 (0.25 - 5.94)	1.21 (0.26 - 5.63)
**Birth weight**	-	-	-
Less than 2500	–	Ref (1)	Ref (1)
2500 - 3999	–	0.22 (0.08 - 0.58)	0.20 (0.08 - 0.50)***
4000+	–	1.67 (1.67 - 4.23)	1.44 (0.58 - 3.58)
**ANC visits**	-	-	-
<8 ANC visits	–	Ref (1)	Ref (1)
8+ ANC visits	–	0.82 (0.07 - 9.04)	0.89 (0.08 - 9.89)
**HIV Results**	-	-	-
HIV negative	–	Ref (1)	Ref (1)
HIV positive	–	3.64 (1.53 - 8.69)	2.42 (1.03 - 5.70)***
**Region**	-	-	-
Central	–	Ref (1)	Ref (1)
Copperbelt	–	0.75 (0.15 - 3.67)	0.56 (0.12 - 2.52)
Eastern	–	0.99 (0.30 - 3.32)	0.82 (0.26 - 2.60)
Luapula	–	1.49 (0.01 - 1.58)	2.21 (0.70 - 6.92)
Muchinga	–	1.67 (0.53 - 5.22)	2.44 (0.83 - 7.18)
Northern	–	0.77 (0.23 - 2.61)	0.97 (0.30 - 3.10)
North Western	–	1.57 (0.45 - 5.43)	1.66 (0.52 - 5.32)
Southern	–	1.17 (0.36 - 3.84)	0.95 (0.32 - 2.84)
Western	–	0.99 (0.28 - 3.55)	1.08 (0.31 - 3.80)
**Community Poverty**	-	-	-
Low	–	–	Ref (1)
Medium	–	–	0.46 (0.19 - 1.17)
High	–	–	0.32 (0.12 - 0.83)
**Community Education**	-	-	-
Low	–	–	Ref (1)
Medium	–	–	1.23 (0.55 - 2.74)
High	–	–	1.15 (0.52 - 2.50)
**Community Employment Status**	-	-	-
Low	–	–	Ref (1)
Medium	–	–	1.34 (0.58 - 3.05)
High	–	–	1.50 (0.74 - 3.06)

Community desired children			-
Low	–	–	Ref (1)
Medium	–	–	1.77 (0.89 - 3.51)
High	–	–	0.45 (0.24 - 0.87)***
-		–	-
Predictive Margins	-	-	-
HIV negative	–	–	0.05 (0.04 - 0.06)***
Hiv positive	–	–	0.1 (0.04 - 0.16)***
Diagnostic test	-	-	-
ICC	0.15	0.17	0.03
AIC	2461.906	603.9776	600.0678
BIC	2473.112	840.4792	878.6141

Predictive margins of neonatal mortality among HIV infected and non-infected mothers in rural Zambia.

Additionally, the study presents a graphical depiction of the predictive probabilities of neonatal mortality. The results demonstrate that neonates born to HIV-infected mothers had a significantly higher mortality rate compared to those born to mothers not infected with HIV, as shown in Fig 5.

Legend. Figure 5: Shows the predictive probabilities of neonatal mortality across rural Zambia among HIV infected and non-HIV infected mothers (2018)

Formatted: Normal (Web), Pattern: Clear (White)

Discussion

This study investigated the spatial variations and predictors of neonatal mortality among HIV-infected and non-infected mothers in rural Zambia, utilizing the 2018 Zambia Demographic Health Survey (ZDHS) dataset. Our analysis revealed distinctive patterns in neonatal mortality across various demographic factors shedding light on the intricate interplay of these factors influencing neonatal mortality survival and highlighting regional disparities in neonatal mortality rates in rural Zambia among HIV infected and non-infected mothers. Notably, neonatal mortality exhibited a

significant association in the baseline characteristics with an increase in maternal age, reaching its peak in the 45 to 49 age group, accounting for 100% among HIV infected mothers compared to 95.21% in non- HIV-infected mothers.

However, utilizing multilevel logistic regression, our study found that only women aged 35 to 39 years had lower odds of experiencing neonatal mortality compared to younger mothers aged 15 to 19 years, regardless of their HIV status albeit that maternal age was not statistically significant. This finding is consistent with evidence showing that maternal age can significantly influence neonatal outcomes, with younger and older maternal age groups carrying unique risks. While adolescent mothers (aged 15–19) are often considered high-risk due to socio-economic vulnerabilities and limited access to healthcare, adulthood presents its own set of challenges, such as physiological and emotional stressors, which can adversely affect neonatal health [27-29].

Several studies highlight that maternal age influences birth outcomes through various mechanisms. Young mothers may have better recovery rates from pregnancy-related stress, while adult women might face life transitions (e.g., balancing education, employment, or childbearing) that could limit their access to quality antenatal care. Moreover, socio-cultural expectations, especially in low-resource settings, may push women to have more children or space them closely, increasing the risk of adverse neonatal outcomes [30].

Furthermore the study examined the relationship between maternal HIV status and neonatal mortality in Zambia, utilizing an adjusted model to account for confounding factors. Initial unadjusted analysis indicated a higher risk of mortality for neonates born to HIV-positive mothers compared to those born to HIV-negative mothers. However, the adjusted model revealed that this association was not statistically significant. These findings align with existing literature, which suggests that while maternal HIV infection is associated with increased neonatal mortality risk, the relationship is complex and influenced by various factors. Research has demonstrated that access to quality healthcare and antiretroviral therapy (ART) plays a crucial role in mitigating risks associated with maternal HIV. ART has been shown to significantly reduce vertical transmission of HIV and improve maternal health outcomes, thus potentially lowering neonatal mortality rates among HIV-positive mothers [31,32].

Additionally, socio-economic conditions can greatly affect healthcare access and adherence to ART, further complicating the relationship between maternal HIV status and neonatal outcomes [33,34]. The uncertainty in the adjusted findings from this study highlights the variability in healthcare access and ART adherence among HIV-positive mothers, which is supported by literature indicating disparities in healthcare utilization and treatment adherence across different socio-economic context [35-37]. This underscores the need for comprehensive interventions that not only address HIV but also improve overall maternal healthcare and socio-economic conditions to enhance neonatal outcomes.

The geospatial distribution of neonatal mortality among HIV-infected and non-infected mothers in rural Zambia encompassed all 10 provinces of the country. The analysis revealed significant spatial disparities in neonatal mortality rates, with notable hotspots identified in the North-Western and Eastern provinces for HIV-infected mothers, and in Muchinga province for non-HIV-infected mothers. This discrepancy in neonatal mortality rates across provinces may stem from multiple factors, including disparities in access to healthcare services, variations in socio-economic conditions, differences in maternal education levels, prevalence of diseases, and cultural practices related to childbirth and infant care [32,33,35,38] . The data emphasizes the dynamic nature of neonatal mortality proportions across Zambia's provinces during the specified period. Recognizing the complexities that contribute to neonatal mortality is crucial for designing targeted interventions aimed at reducing mortality rates and improving maternal and child health outcomes nationwide [31,36].

Our multilevel model revealed that female neonates exhibited a lower likelihood of neonatal mortality, indicating that male neonates were more vulnerable to mortality within the first month of life compared to their female counterparts. These findings align with prior research indicating a higher mortality rate among boys compared to girls [39,40]. Furthermore the findings indicate that maternal education significantly influences neonatal mortality, with primary education serving as a protective factor. Mothers with primary education are associated with 88% lower odds of neonatal death compared to those with no education. While secondary or tertiary education shows a similar protective trend (AOR: 0.20, 95% CI: 0.02–1.72), it does not reach statistical significance ($p = 0.143$). These results suggest that even basic education equips mothers with essential

knowledge to improve neonatal health outcomes, reinforcing the need to promote educational access among women in rural areas.

Recent findings highlight the strong impact of maternal education on neonatal and child mortality. A systematic review and meta-analysis published in the lancet found a clear dose-response relationship: mothers with 12 years of education (equivalent to secondary school completion) were associated with a 31% reduction in under-5 mortality compared to those with no education. Additionally, each additional year of maternal schooling reduced child mortality by approximately 3%. The study emphasizes that maternal education remains a stronger predictor of child survival compared to paternal education, underscoring its transgenerational benefits for child health [38] These findings align with recommendations in the WHO's 2023 progress report on maternal and newborn health, which calls for investments in education as part of strategies to reduce neonatal mortality. The report highlights the need for integrated efforts, including better healthcare access and education, to meet global health goals [41].

Formatted: Font: (Default) Times New Roman

Furthermore our study emphasizes the multifaceted nature of factors influencing neonatal mortality, including maternal age, neonate sex, maternal HIV status, and duration of breastfeeding, place of delivery and Region (North Western province). This intricate web of variables highlights the need for a shift in focus within current mother and child health programs to incorporate activities that empower women to make informed decisions regarding the timing of childbirth, monitor their HIV status, duration of breastfeeding and consider the birthweight and sex of their child. Additionally, a systematic review and meta-analysis conducted in sub-Saharan Africa revealed that rural residence significantly contributes to high neonatal mortality rates [42]. Our study's novel findings offer valuable insights previously unexplored, illuminating the intricate landscape of neonatal mortality in rural Zambia. Recognizing the distinct patterns of neonatal mortality between HIV-infected and non-infected mothers is crucial for implementing tailored interventions that enhance maternal and child health outcomes within these communities.

This study investigated the spatial patterns and predictors of neonatal mortality among HIV-infected and non-infected mothers in rural Zambia, utilizing the 2018 Zambia Demographic Health Survey (ZDHS) dataset. Our analysis revealed distinctive patterns in neonatal mortality across various demographic factors shedding light on the intricate interplay of these factors influencing

Formatted: Space Before: 0 pt, After: 8 pt

neonatal mortality survival and highlighting regional disparities in neonatal mortality rates in rural Zambia among HIV infected and non-infected mothers. Notably, neonatal mortality exhibited a significant association with an increase in maternal age, reaching its peak in the 45 to 49 age group, accounting for 100% among HIV infected mothers compared to 95.21% in non- HIV-infected mothers. Furthermore, the geospatial findings revealed that the region with the highest rates of neonatal mortality consistently for the three time periods of the demographic health surveys was Western Province; Lusaka, Eastern, and Muchinga fluctuated, and Copperbelt maintained second place throughout the periods.

The study's findings regarding the association between maternal age and neonatal mortality are consistent with previous research, which has consistently demonstrated a heightened risk of neonatal complications with increasing maternal age [22,23]. This alignment underscores the well-established understanding that rates of adverse pregnancy outcomes, including neonatal mortality, tend to rise significantly as maternal age advances [24]. These consistent findings emphasize the crucial importance of educating women about the potential risks associated with delayed motherhood [25]. Conversely, a study in Brazil focusing solely on the impact of maternal age on neonatal death found elevated mortality rates among adolescent mothers compared to their older counterparts [26]. However, our study, employing multilevel logistic regression, revealed that women aged 45 to 49, regardless of HIV infection status, exhibited increased odds of neonatal mortality compared to mothers aged 15 to 19 years. This finding contrasts with research conducted in Bangladesh, where women aged 19 years and above showed higher odds of neonatal mortality compared to those younger than 19 [27-29].

Furthermore, our investigation revealed that female neonates exhibited a lower likelihood of neonatal mortality, indicating that male neonates were more vulnerable to mortality within the first month of life compared to their female counterparts. These findings align with prior research indicating a higher mortality rate among boys compared to girls [30,31]. Additionally, our results indicated that neonates with normal birth weight had a decreased likelihood of mortality compared to those with low birth weight, consistent with studies conducted in Kenya [32] and Nepal [33].

Furthermore, our study emphasized maternal HIV status on neonatal mortality in rural Zambia. The analysis of predictive margins revealed that neonates born to HIV-infected mothers faced an

Formatted: Space Before: Auto, After: Auto

increased probability of mortality compared to those born to non-infected mothers. Multiple studies have highlighted alarmingly high mortality rates among HIV-exposed children [34,35]. Studies have shown that HIV exposure is associated with low birth weight, prematurity, and a higher proportion of infants being small for gestational age [36-38]. Moreover, chronic immune activation resulting from maternal HIV infection impacts infant immunology levels, and HIV-exposed but uninfected children often possess lower levels of maternally transferred antibodies at birth [35].

Our study investigated geospatial patterns of neonatal mortality among HIV-infected and non-infected mothers of rural Zambia across 10 provinces of Zambia. Nationally, neonatal mortality levels exhibited spatial variation, with hotspots identified in North-western and Eastern provinces among HIV-infected mothers, and Muchinga province among non-HIV-infected mothers. This discrepancy in neonatal mortality rates across provinces may stem from multiple factors, including disparities in access to healthcare services, variations in socio-economic conditions, differences in maternal education levels, prevalence of diseases, and cultural practices related to childbirth and infant care. The data emphasizes the dynamic nature of neonatal mortality proportions across Zambia's provinces during the specified period. Understanding the underlying factors contributing to these regional disparities is paramount for formulating targeted and effective interventions aimed at reducing neonatal mortality rates and improving maternal and child health outcomes nationwide.

The Sustainable Development Goals (SDGs) have set a global target to reduce neonatal mortality rates to no more than 12 infant deaths per 1,000 live births by 2030 [39]. However, achieving this target faces significant challenges, particularly in regions such as sub-Saharan Africa, where numerous factors contribute to elevated neonatal mortality levels. According to UNICEF, a lot of countries are at risk of missing the SDG target on neonatal mortality than on under-five mortality [40]. Moreover, our study findings emphasizes the multifaceted nature of factors influencing neonatal mortality, including maternal age, neonate sex, birthweight, maternal HIV status, and community desired number of children. This intricate web of variables highlights the need for a shift in focus within current mother and child health programs to incorporate activities that empower women to make informed decisions regarding the timing of childbirth, monitor their HIV status, consider the birthweight and sex of their child, among other crucial factors. Moreover, a

Commented [WU1]: • Chileshe, M., & Kasonde, M. (2022). Regional disparities in neonatal mortality in Zambia: A call for targeted interventions. *Zambia Medical Journal*.
• Brown, T., & Green, D. (2019). The impact of socio-economic factors on maternal and neonatal health outcomes in sub-Saharan Africa. *Global Health Action*.
• Anderson, K. L., & Taylor, B. (2021). Healthcare access and neonatal mortality: A comprehensive review of Zambia's provinces. *BMC Public Health*.

Formatted: Font: (Default) Times New Roman

Formatted: Font: (Default) Times New Roman, 12 pt

systematic review study and meta-analysis conducted in sub-Saharan Africa revealed that rural residence was a significant contributing factor to high neonatal mortality rates [41]. Our study's novel findings offer valuable insights previously unexplored, illuminating the intricate landscape of neonatal mortality in rural Zambia. Recognizing the distinct patterns of neonatal mortality between HIV-infected and non-infected mothers is crucial for tailored interventions and enhancing maternal and child health outcomes within these communities.

Study strength and study Limitations

This study benefits from the utilization of national data, offering a representative sample of neonates, thus enabling generalizability to this specific demographic. However, certain limitations must be acknowledged. The study relies on the 2018 Zambia Demographic and Health Survey (ZDHS) dataset, employing a cross-sectional design, which indicates correlation rather than causation between the outcome and individual or contextual factors. Additionally, the utilization of contextual and community-level factors from the ZDHS may not fully capture the community experience. Moreover, potential recall bias exists, with mothers possibly misreporting crucial information like their child's age at death, birth order, and birth weight. To address this, rigorous data cleaning procedures were implemented to identify and rectify inconsistencies or outliers.

Policy recommendation

To effectively address the disparities in neonatal mortality rates among HIV-infected and non-infected mothers in rural Zambia, it is essential to implement a multifaceted approach that focuses on improving access to quality healthcare services, particularly in the identified hotspot regions of North-Western, Eastern, and Muchinga provinces. This can be achieved by enhancing healthcare infrastructure and resources, ensuring the availability of essential maternal and child health services, and increasing the reach of antiretroviral therapy (ART) programs for HIV-positive mothers. Additionally, targeted educational campaigns should be developed to raise awareness about safe childbirth practices, infant care, and the importance of maternal health, particularly in communities with low maternal education levels. Collaborating with local stakeholders, including traditional leaders and community health workers, can foster culturally sensitive interventions that resonate with local practices and beliefs. Furthermore, addressing socio-economic determinants,

such as poverty and access to education, will be crucial in creating an enabling environment for improving maternal and child health outcomes across all provinces.

Zambia has demonstrated a steadfast commitment to improving neonatal survival through targeted interventions from 2000 to 2017, focusing on bolstering healthcare infrastructure, community outreach programs, and healthcare worker capacity building. Fluctuations in neonatal mortality across provinces are likely influenced by the implementation of key programs like maternal and perinatal death reviews, essential newborn care, and Kangaroo mother care. To address rural Zambia's neonatal mortality, a comprehensive approach is recommended. This includes strengthening healthcare infrastructure, enhancing community outreach, providing ongoing training for healthcare workers, improving maternal education, and tailoring interventions to address regional disparities based on factors like access to healthcare and socio-economic conditions. These efforts signify Zambia's dedication to improving maternal and child health outcomes and enhancing neonatal survival rates.

Conclusion

In conclusion, this study highlights significant geospatial variations in neonatal mortality rates among HIV-infected and non-infected mothers across rural Zambia, emphasizing the need for targeted interventions to address these disparities. The findings reveal that regional differences in access to healthcare, socio-economic conditions, maternal education levels, and cultural practices significantly influence neonatal outcomes. Addressing these multifaceted factors is crucial for improving maternal and child health in Zambia. By enhancing healthcare access, increasing the availability of antiretroviral therapy, and implementing culturally sensitive educational campaigns, stakeholders can work collaboratively to reduce neonatal mortality rates. Ultimately, a comprehensive approach that considers the unique challenges faced by different provinces will be essential for achieving equitable health outcomes and promoting the well-being of mothers and their infants throughout the country. In conclusion, the findings in this study provide valuable insights into the spatial patterns and determinants of neonatal mortality among HIV-infected and non-infected mothers in rural Zambia. The findings reveal distinct patterns of neonatal mortality across various demographic factors, emphasizing the complex interplay of these factors and highlighting regional disparities in neonatal mortality rates. Notably, maternal age, sex of the neonate, birthweight, HIV status of the mother, and community's desired number of children emerged as significant predictors of neonatal mortality. Geospatial analysis identified hotspots of neonatal mortality in certain provinces, underscoring the need for targeted interventions tailored

Formatted: Font: (Default) Times New Roman

to regional contexts. Furthermore, the study emphasizes the need for strengthening healthcare infrastructure, promoting community outreach programs, enhancing healthcare worker training, focusing on maternal education, and addressing regional disparities. These recommendations aim to improve maternal and child health outcomes and contribute to the achievement of Sustainable Development Goals (SDGs) targets.

Acknowledgement

Many thanks to the Zambia Statistical Agency (ZSA) and the DHS program for granting permission to utilize the 2018 ZDHS.

Dedication

This paper is dedicated, with love and reverence, to the cherished memory of Wise Mwila Katumbo.

Reference

1. Newborn Mortality (who.int) https://www.who.int/news-room/fact-sheets/detail/levels-and-trends-in-child-mortality-report-2021
2. Hug L, Sharrow D, Zhong K, You D. Levels & trends in child mortality. New York, USA: United Nations Children's Fund. 2017:1-40.
3. UN Inter-Agency Group for Child Mortality Estimation. UNICEF. WHO. Levels & Trends in Child Mortality Estimation Child Mortality. UNICEF; New York, NY, USA: 2020.
4. Akombi BJ, Renzaho AM. Perinatal mortality in sub-Saharan Africa: a meta-analysis of demographic and health surveys. Annals of global health. 2019;85(1).
5. Tekelab T, Chojenta C, Smith R, Loxton D. The impact of antenatal care on neonatal mortality in sub-Saharan Africa: A systematic review and meta-analysis. PloS one. 2019 Sep 13;14(9):e0222566.
6. Waiswa P. Understanding newborn care in Uganda: Towards future interventions. Karolinska Institutet (Sweden); 2010.
7. Kananura RM, Tetui M, Mutebi A, Bua JN, Waiswa P, Kiwanuka SN, Ekirapa-Kiracho E, Makumbi F. The neonatal mortality and its determinants in rural communities of Eastern Uganda. Reproductive health. 2016 Dec;13:1-9.

Formatted: Font: (Default) Times New Roman, 12 pt

8. UNICEF. UNICEF Data Warehouse. Available from: https://data.unicef.org/resources/data_explorer/unicef_f/. Accessed 5 October 2021.
9. Zambia Statistics Agency ZSA, Ministry of Health MOH, University Teaching Hospital Virology Laboratory U-V, Icf. Zambia Demographic and Health Survey 2018. Lusaka, Zambia: ZSA, MOH, UTH-VL and ICF; 2020.
10. Kc A, Jha AK, Shrestha MP, Zhou H, Gurung A, Thapa J, Budhathoki SS. Trends for neonatal deaths in Nepal (2001–2016) to project progress towards the SDG target in 2030, and risk factor analyses to focus action. Maternal and child health journal. 2020 Feb;24:5-14.
11. Grady SC, Frake AN, Zhang Q, Bene M, Jordan DR, Vertalka J, Dossantos TC, Kadhim A, Namanya J, Pierre LM, Fan Y. Neonatal mortality in East Africa and West Africa: a geographic analysis of district-level demographic and health survey data. Geospatial health. 2017 May 26;12(1).
12. Tembo D, Abobo FD, Kaonga P, Jacobs C, Bessing B. Risk factors associated with neonatal mortality among neonates admitted to neonatal intensive care unit of the University Teaching Hospital in Lusaka. Scientific Reports. 2024 Mar 4;14(1):1-2.
13. Kamanga A, Ngosa L, Aladesanmi O, Zulu M, McCarthy E, Choba K, Nyirenda J, Chizuni C, Mwiche A, Storey A, Shakwelele H. Reducing maternal and neonatal mortality through integrated and sustainability-focused programming in Zambia. PLOS Global Public Health. 2022 Dec 14;2(12):e0001162.
14. Guinsburg R, Sanudo A, Kiffer CR, Marinonio AS, Costa-Nobre DT, Areco KN, Kawakami MD, Miyoshi MH, Bandiera-Paiva P, Balda RD, Konstantyner T. Annual trend of neonatal mortality and its underlying causes: population-based study–São Paulo State, Brazil, 2004–2013. BMC pediatrics. 2021 Jan 26;21(1):54.
15. Norris M, Klabbers G, Pembe AB, Hanson C, Baker U, Aung K, Mmweteni M, Mfaume RS, Beňová L. A growing disadvantage of being born in an urban area? Analysing urban–rural disparities in neonatal mortality in 21 African countries with a focus on Tanzania. BMJ Global Health. 2022 Jan 1;7(1):e007544.
16. Shumba S, Moonga V, Miyoba TO, Jere S, Nkonde JM, Mumba P (2024) Socio-economic disparities and predictors of fertility among adolescents aged 15 to 19 in Zambia: Evidence from the Zambia Demographic and Health Survey (2018). PLOS Glob Public Health 4(3): e0002739. https://doi.org/10.1371/journal.pgph.0002739
17. Kujala S, Waiswa P, Kadobera D, Akuze J, Pariyo G, Hanson C. Trends and risk factors of stillbirths and neonatal deaths in Eastern Uganda (1982–2011): a cross-sectional, population-based study. Tropical Medicine & International Health. 2017 Jan;22(1):63-73.
18. Saloio CÁ, Morais Neto OL, Gonçalves DA, Bessa HE, Coelho Júnior JP, Afonso MS, Carvalho SR. Magnitude and determinants of neonatal and postneonatal mortality in Goiânia, Goiás, Brazil: a retrospective cohort study, 2012. Epidemiologia e Serviços de Saúde. 2020 Nov 16;29:e2020132.
19. Shumba S, Fwemba I, Kaymba V (2024) Spatial-temporal patterns and predictors of timing and inadequate antenatal care utilization in Zambia: A Generalized Linear Mixed Model (GLMM) investigation from 1992 to 2018. PLOS Glob Public Health 4(10): e0003213. https://doi.org/10.1371/journal.pgph.0003213
20. Miyoshi Y, Matsubara K, Takata N, Oka Y. Baby survival in Zambia: stillbirth and neonatal death in a local hospital setting. BMC pregnancy and childbirth. 2019 Dec;19:1-6.

Formatted: Font: (Default) Times New Roman, 12 pt

21. Saleem S, Naqvi F, McClure EM, Nowak KJ, Tikmani SS, Garces AL, Hibberd PL, Moore JL, Nolen TL, Goudar SS, Kumar Y. Neonatal deaths in infants born weighing≥ 2500 g in low and middle-income countries. Reproductive Health. 2020 Nov;17:1-4.
22. Shumba S, Jacobs C, Kapembwa M, Miyoba TO, Musonda NC. Spatial analysis and associated factors of underweight, overweight and obesity among non-pregnant women of reproductive age in Zambia. African Health Sciences. 2024 Oct 6;24(3):292-301.
23. Shiferaw K, Mengistie B, Gobena T, Dheresa M, Seme A. Neonatal mortality rate and its Determinants: A community–based panel study in Ethiopia. Frontiers in Pediatrics. 2022 May 23;10:875652.
24. Mosley WH, Chen LC. An analytical framework for the study of child survival in developing countries. 1984. Bull World Health Organ. 2003;81(2):140-5. PMID: 12756980; PMCID: PMC2572391.
25. https://dhsprogram.com/topics/wealth-index/Wealth-Index-Construction.cfm.
26. Snijders TA, Bosker R. Multilevel analysis: An introduction to basic and advanced multilevel modeling.2011
27. UNICEF. (2023). *The State of the World's Children 2023: For Every Child, Health, Education, and Protection*.
28. WHO. (2022). *Maternal and Neonatal Health Guidelines*.
29. Shumba S, Moonga V, Miyoba TO, Jere S, Nkonde JM, Mumba P. Socio-economic disparities and predictors of fertility among adolescents aged 15 to 19 in Zambia: Evidence from the Zambia demographic and health survey (2018). PLOS Global Public Health. 2024 Mar 27;4(3):e0002739.
30. Kota K, Chomienne MH, Geneau R, Yaya S. Socio-economic and cultural factors associated with the utilization of maternal healthcare services in Togo: a cross-sectional study. Reproductive Health. 2023 Jul 25;20(1):109.
31. Smith, J. A., & Jones, R. L. (2020). The impact of maternal ART on neonatal outcomes: A systematic review. Journal of Maternal-Fetal & Neonatal Medicine.
32. Brown, T., & Green, D. (2019). Socioeconomic factors affecting neonatal mortality in HIV-positive mothers. Global Health Action.
33. Anderson, K. L., & Taylor, B. (2021). Access to healthcare and its impact on maternal and neonatal health in sub-Saharan Africa. *BMC Public Health*.
34. Ngwenya, N., & Makhubele, B. (2018). The role of socio-economic status in healthcare access and ART adherence among HIV-positive women. *AIDS Care*.
35. Chileshe, M., & Kasonde, M. (2022). Disparities in healthcare access for HIV-positive mothers in Zambia: Implications for neonatal health. *Zambia Medical Journal*.
36. Tende, M., & Mumba, C. (2020). The influence of socio-economic factors on health outcomes for HIV-positive mothers: A Zambian perspective. *African Journal of Health Sciences*.
37. Tembo D, Abobo FD, Kaonga P, Jacobs C, Bessing B. Risk factors associated with neonatal mortality among neonates admitted to neonatal intensive care unit of the University Teaching Hospital in Lusaka. Scientific Reports. 2024 Mar 4;14(1):5231.
38. Shumba S, Jacobs C, Kapembwa M, Miyoba TO, Musonda NC. Spatial analysis and associated factors of underweight, overweight and obesity among non-pregnant women of reproductive age in Zambia. African Health Sciences. 2024 Oct 6;24(3):292-301.
39. Hug L, Alexander M, You D, Alkema L. National, regional, and global levels and trends in neonatal mortality between 1990 and 2017, with scenario-based projections to 2030: a systematic analysis. The Lancet Global Health. 2019 Jun 1;7(6):e710-20.

Formatted: Font: (Default) Times New Roman, 12 pt

Formatted: Font: (Default) Times New Roman, 12 pt

40. UNICEF. UNICEF Data Warehouse. Available from: https://data.unicef.org/resources/data_explorer/unicef_f/. Accessed 5 October 2021.
41. WHO. Improving maternal and newborn health and survival and reducing stillbirth - Progress report 2023. https://www.who.int/publications/i/item/9789240073678
42. Bech CM, Stensgaard CN, Lund S, Holm-Hansen C, Brok JS, Nygaard U, Poulsen A. Risk factors for neonatal sepsis in Sub-Saharan Africa: a systematic review with meta-analysis. BMJ open. 2022 Sep 1;12(9):e054491.

1. Newborn Mortality (who.int) https://www.who.int/news-room/fact-sheets/detail/levels-and-trends-in-child-mortality-report-2021
2. Hug L, Sharrow D, Zhong K, You D. Levels & trends in child mortality. New York, USA: United Nations Children's Fund. 2017:1-40.
3. UN Inter-Agency Group for Child Mortality Estimation. UNICEF. WHO. Levels & Trends in Child Mortality Estimation Child Mortality. UNICEF; New York, NY, USA: 2020.
4. Akombi BJ, Renzaho AM. Perinatal mortality in sub-Saharan Africa: a meta-analysis of demographic and health surveys. Annals of global health. 2019;85(1).
5. Tekelab T, Chojenta C, Smith R, Loxton D. The impact of antenatal care on neonatal mortality in sub-Saharan Africa: A systematic review and meta-analysis. PloS one. 2019 Sep 13;14(9):e0222566.
6. Waiswa P. Understanding newborn care in Uganda: Towards future interventions. Karolinska Institutet (Sweden); 2010.
7. Kananura RM, Tetui M, Mutebi A, Bua JN, Waiswa P, Kiwanuka SN, Ekirapa-Kiracho E, Makumbi F. The neonatal mortality and its determinants in rural communities of Eastern Uganda. Reproductive health. 2016 Dec;13:1-9.
8. UNICEF. UNICEF Data Warehouse. Available from: https://data.unicef.org/resources/data_explorer/unicef_f/. Accessed 5 October 2021.
9. Zambia Statistics Agency ZSA, Ministry of Health MOH, University Teaching Hospital Virology Laboratory U-V, Icf. Zambia Demographic and Health Survey 2018. Lusaka, Zambia: ZSA, MOH, UTH-VL and ICF; 2020.
10. Kc A, Jha AK, Shrestha MP, Zhou H, Gurung A, Thapa J, Budhathoki SS. Trends for neonatal deaths in Nepal (2001–2016) to project progress towards the SDG target in 2030, and risk factor analyses to focus action. Maternal and child health journal. 2020 Feb;24:5-14.
11. Grady SC, Frake AN, Zhang Q, Bene M, Jordan DR, Vertalka J, Dossantos TC, Kadhim A, Namanya J, Pierre LM, Fan Y. Neonatal mortality in East Africa and West Africa: a geographic analysis of district-level demographic and health survey data. Geospatial health. 2017 May 26;12(1).
12. Tembo D, Abobo FD, Kaonga P, Jacobs C, Bessing B. Risk factors associated with neonatal mortality among neonates admitted to neonatal intensive care unit of the University Teaching Hospital in Lusaka. Scientific Reports. 2024 Mar 4;14(1):1-2.

Formatted: Font: (Default) Times New Roman, 12 pt

Formatted: Font: (Default) Times New Roman, 12 pt

Formatted: Font: (Default) Times New Roman, 12 pt

Formatted: Normal, Indent: Left: 0.25", No bullets or numbering

Formatted: Font: (Default) Times New Roman, 12 pt

Formatted: Normal, Left, No bullets or numbering

Formatted: Font: (Default) Times New Roman, 12 pt

13. Kamanga A, Ngosa L, Aladesanmi O, Zulu M, McCarthy E, Choba K, Nyirenda J, Chizuni C, Mwiche A, Storey A, Shakwelele H. Reducing maternal and neonatal mortality through integrated and sustainability-focused programming in Zambia. PLOS Global Public Health. 2022 Dec 14;2(12):e0001162.

14. Guinsburg R, Sanudo A, Kiffer CR, Marinonio AS, Costa-Nobre DT, Areco KN, Kawakami MD, Miyoshi MH, Bandiera-Paiva P, Balda RD, Konstantyner T. Annual trend of neonatal mortality and its underlying causes: population-based study–São Paulo State, Brazil, 2004–2013. BMC pediatrics. 2021 Jan 26;21(1):54.

15. Norris M, Klabbers G, Pembe AB, Hanson C, Baker U, Aung K, Mmweteni M, Mfaume RS, Beňová L. A growing disadvantage of being born in an urban area? Analysing urban–rural disparities in neonatal mortality in 21 African countries with a focus on Tanzania. BMJ Global Health. 2022 Jan 1;7(1):e007544.

16. Kujala S, Waiswa P, Kadobera D, Akuze J, Pariyo G, Hanson C. Trends and risk factors of stillbirths and neonatal deaths in Eastern Uganda (1982–2011): a cross-sectional, population-based study. Tropical Medicine & International Health. 2017 Jan;22(1):63-73.

17. Saloio CÁ, Morais Neto OL, Gonçalves DA, Bessa HE, Coelho Júnior JP, Afonso MS, Carvalho SR. Magnitude and determinants of neonatal and postneonatal mortality in Goiânia, Goiás, Brazil: a retrospective cohort study, 2012. Epidemiologia e Serviços de Saúde. 2020 Nov 16;29:e2020132.

18. Miyoshi Y, Matsubara K, Takata N, Oka Y. Baby survival in Zambia: stillbirth and neonatal death in a local hospital setting. BMC pregnancy and childbirth. 2019 Dec;19:1-6.

19. Saleem S, Naqvi F, McClure EM, Nowak KJ, Tikmani SS, Garces AL, Hibberd PL, Moore JL, Nolen TL, Goudar SS, Kumar Y. Neonatal deaths in infants born weighing≥ 2500 g in low and middle-income countries. Reproductive Health. 2020 Nov;17:1-4.

20. Shiferaw K, Mengistie B, Gobena T, Dheresa M, Seme A. Neonatal mortality rate and its Determinants: A community–based panel study in Ethiopia. Frontiers in Pediatrics. 2022 May 23;10:875652.

21. https://dhsprogram.com/topics/wealth-index/Wealth-Index-Construction.cfm.

22. Kahveci B, Melekoglu R, Evruke IC, Cetin C. The effect of advanced maternal age on perinatal outcomes in nulliparous singleton pregnancies. BMC pregnancy and childbirth. 2018 Dec;18:1-7.

23. Fonseca MJ, Santos F, Afreixo V, Silva IS, do Céu Almeida M. Does induction of labor at term increase the risk of cesarean section in advanced maternal age? A systematic review and meta-analysis. European Journal of Obstetrics & Gynecology and Reproductive Biology. 2020 Oct 1;253:213-9.

Formatted: Font: (Default) Times New Roman, 12 pt

Formatted: Highlight

Formatted: Font: (Default) Times New Roman, 12 pt

24. Nyongesa P, Ekhaguere OA, Marete I, Tenge C, Kemoi M, Bann CM, Bucher SL, Patel AB, Hibberd PL, Naqvi F, Saleem S. Maternal age extremes and adverse pregnancy outcomes in low-resourced settings. Frontiers in Global Women's Health. 2023;4.

25. Vandekerckhove M, Guignard M, Civadier MS, Benachi A, Bouyer J. Impact of maternal age on obstetric and neonatal morbidity: a retrospective cohort study. BMC pregnancy and childbirth. 2021 Dec;21:1-7.

26. Correa-de-Araujo R, Yoon SS. Clinical outcomes in high-risk pregnancies due to advanced maternal age. Journal of Women's Health. 2021 Feb 1;30(2):160-7.

27. Huda TM, Tahsina T, Arifeen SE, Dibley MJ. The importance of intersectoral factors in promoting equity-oriented universal health coverage: a multilevel analysis of social determinants affecting neonatal infant and under-five mortality in Bangladesh. Global health action. 2016 Dec 1;9(1):29741.

28. Jehan I, Harris H, Salat S, Zeb A, Mobeen N, Pasha O, McClure EM, Moore J, Wright LL, Goldenberg RL. Neonatal mortality, risk factors and causes: a prospective population-based cohort study in urban Pakistan. Bulletin of the world Health Organization. 2009 Feb;87(2):130-8.

29. Chen XK, Wen SW, Fleming N, Yang Q, Walker MC. Increased risks of neonatal and postneonatal mortality associated with teenage pregnancy had different explanations. Journal of Clinical Epidemiology. 2008 Jul 1;61(7):688-94.

30. Abdellatif M, Ahmed M, Bataclan MF, Khan AA, Al Battashi A, Al Maniri A. The patterns and causes of neonatal mortality at a tertiary hospital in oman. Oman medical journal. 2013 Nov;28(6):422.

31. Thammanna PS, Sridhar PV, Sandeep M. Morbidity pattern and hospital outcome of neonates admitted in a tertiary care teaching hospital, Mandya. International Journal of Scientific Study. 2015;3(6):126-9.

32. Imbo AE, Mbuthia EK, Ngotho DN. Determinants of neonatal mortality in Kenya: evidence from the Kenya demographic and health survey 2014. International Journal of Maternal and Child Health and AIDS. 2021;10(2):287.

33. Neupane D, Dawson P, Houston R, Dhakal L, Sharma J, Gargi KC, Lagos C, Khanal V, Mishra SR, Kallestrup P. Lower mortality is observed among low birth weight young infants who have received home-based care by female community health volunteers in rural Nepal. BMC pregnancy and childbirth. 2017 Dec;17:1-7.

34. Taha TE, Dadabhai SS, Sun J, Rahman MH, Kumwenda J, Kumwenda N. Child mortality levels and trends by HIV status in Blantyre, Malawi: 1989–2009. JAIDS Journal of Acquired Immune Deficiency Syndromes. 2012 Oct 1;61(2):226-34.

35. Obeagu EI, Obeagu GU. Neonatal Outcomes in Children Born to Mothers with Severe Malaria, HIV, and Transfusion History: A Review. Elite Journal of Nursing and Health Science. 2024;2(3):38-58.

Formatted: Highlight

Formatted: Font: (Default) Times New Roman, 12 pt

Formatted: Highlight

Formatted: Font: (Default) Times New Roman, 12 pt

36. Xiao PL, Zhou YB, Chen Y, Yang MX, Song XX, Shi Y, Jiang QW. Association between maternal HIV infection and low birth weight and prematurity: a meta-analysis of cohort studies. BMC pregnancy and childbirth. 2015 Dec;15:1-1.

37. Sania A, Smith ER, Manji K, Duggan C, Masanja H, Kisenge R, Msamanga G, Urassa W, Fawzi W. Neonatal and infant mortality risk associated with preterm and small for gestational age births in Tanzania: individual level pooled analysis using the intergrowth standard. The Journal of pediatrics. 2018 Jan 1;192:66-72.

38. Twabi HS, Manda SO, Small DS. Assessing the effects of maternal HIV infection on pregnancy outcomes using cross-sectional data in Malawi. BMC Public Health. 2020 Jun 22;20(1):974.

39. Hug L, Alexander M, You D, Alkema L. National, regional, and global levels and trends in neonatal mortality between 1990 and 2017, with scenario-based projections to 2030: a systematic analysis. The Lancet Global Health. 2019 Jun 1;7(6):e710-20.

40. UNICEF. UNICEF Data Warehouse. Available from: https://data.unicef.org/resources/data_explorer/unicef_f/. Accessed 5 October 2021.

41. Bech CM, Stensgaard CN, Lund S, Holm-Hansen C, Brok JS, Nygaard U, Poulsen A. Risk factors for neonatal sepsis in Sub-Saharan Africa: a systematic review with meta-analysis. BMJ open. 2022 Sep 1;12(9):e054491.

Formatted: Font: (Default) Times New Roman, 12 pt

PLoS Global Public Health

Dear Editor,

We appreciate the insightful comments and constructive feedback provided by the reviewers on our manuscript, *"Spatial Variations and Predictors of Neonatal Mortality among HIV-Infected and Non-Infected Mothers in Rural Zambia."* Their observations have significantly improved the quality of our study. Below, we present a point-by-point response detailing how we have addressed each concern.

Reviewer 1

The authors attempt to provide an understanding of geospatial variability in HIV cases and deaths in Zambia and also understand the most significant predictors. The study lacks flow and the methods are poorly written. Significant improvements are needed to clarify the key message and the purpose of the study. The title is overly complex and would benefit from reorientation based on clearly defined objectives, results, and conclusions. The authors often repeat statements already presented in the tables. For instance, the discussion on geospatial patterns and predictors of neonatal mortality implies that geospatial mapping is a key component, which is not adequately reflected in the study itself. There are also a lot of repetitive words and sentences and authors can benefit from a revision that enhances readability. For example, Authors keep referring to children – amend to neonates

Ans: Corrected

Title: Too crowded and not clear enough.

Ans: Refined (see page 1)

Abstract:

Needs to be more concise and is poorly written. For example, the first two sentences in the background below don't belong in abstract.

"*Neonatal mortality is the death of a live-born infant within the first 28 completed days of life. Neonatal mortality remains a major public health concern in most African countries, with the Sub-Saharan region being the highest contributor at 27 deaths per 1000 live births, accounting for 43% of the total number of deaths*."

Ans: Refined (see page 2)

Methods

More specifically the methods section lacks clarity on why spefic variables were chosen, conceptually how they affect the outcomes, the rationale for the methods including choice of mixed effects model.

Ans: Done, an explanation has been included in the Dependent and independent variables Section see page 4. Also we have added a conceptual framework to support where we obtained the variables from.

On Community level variables; *Community-level variables in this study were derived by aggregating individual-level data into clusters and incorporated community poverty, community education, community knowledge of family planning (FP) methods, and place of residence.*

Ans: We have actually removed them because we didn't have enough power to actually have more variables in the model.

Data Analysis

For descriptive purposes, frequencies and percentages were computed for categorical variables. To determine the association between the outcome variable (neonatal mortality) and the categorical variables, the Uncorrelated Design Based Chi-square test (Rao – Scott Chi-square test) was used. The multilevel mixed-effect logistic regression model was employed to determine the factors affecting newborn mortality. Additionally, the study used an investigator-led approach, all variables were selected from a wide range of literature. The Intra-Class Correlation (ICC), Akaike Information Criteria (AIC), and the Bayesian Information Criteria (BIC) were sufficiently explored to select the best-fit model. Stata version 14.2 was used for analysis.

Not clear what analysis was done. The authors alude they did AIC and BIC to perform model selection; but was the model/variable (at this point it is very confusing as to what the paper is all about) selection fit into a final model? And why was it done?

Ans: What Analysis Was Done?

- **Descriptive Analysis: Frequencies and percentages were computed for categorical variables to provide a summary of the data.**
- **Association Testing: The Rao–Scott Chi-square test was used to determine the association between neonatal mortality (the outcome) and categorical variables, accounting for the survey design.**
- **Multilevel Mixed-Effects Logistic Regression: This model was employed to analyze the factors influencing neonatal mortality, accounting for individual-level and community-level variations. The mixed-effects model is appropriate because the dataset has a hierarchical structure (individuals nested within communities).**

We used the Akaike Information Criterion (AIC) and Bayesian Information Criterion (BIC) for model selection to identify the most parsimonious model that balances goodness of fit with complexity. AIC rewards goodness of fit but penalizes the number of parameters, while BIC imposes a stronger penalty for model complexity. These criteria help us ensure the final model avoids overfitting by retaining only variables that meaningfully contribute to explaining neonatal mortality. This process ensures robust results aligned with the study's focus on key predictors.

In description of Multilevel logistic regression, was it a multivariable model? what were the levels in the hierarchy and what were the fixed effects, what were the random effects? Below is an example

In the description of the Multilevel Logistic Regression model, the following points need to be addressed for clarity:

Multivariable Model: Confirm if the model included multiple predictors.

- Levels in the Hierarchy: Clearly define the hierarchical structure:

- Level 1: Individual level (e.g., neonates).

- Level 2: Cluster level (e.g., health facilities or geographical regions).

Ans: Yes, the multivariable model did include multiple predictors to explore the factors associated with neonatal mortality. These predictors spanned individual characteristics such as maternal age, education, and birthweight, along with cluster-level factors like place of delivery and regional location.

Regarding the hierarchical structure, we used a multilevel model to account for the nested nature of the data:

- **Level 1: Individual-level predictors (e.g., characteristics of neonates and their mothers)**
- **Level 2: Cluster-level variables (e.g., geographical regions or enumeration areas)**

Fixed Effects: Specify the predictors considered as fixed effects, such as:

- Socio-demographic factors (e.g., maternal age, education level, household income).

- Health-related variables (e.g., prenatal care visits, birth weight, delivery method).

Ans: Noted they have been specified see page 4

Random Effects: Identify the random effects, which account for variability at the cluster level:

- Random intercepts (e.g., differences in neonatal mortality rates across clusters).

- Random slopes for some predictors, if applicable (e.g., varying effects of prenatal care visits across different regions).

Providing this detailed information will enhance the clarity and comprehensiveness of the methods section.

Ans: Been provided check on page page 13

What was the outcome and how were the variables in the final model(s) chosen?

Ans: the outcome variable in the model was neonatal mortality coded as dead or alive. The best fit model was selected through the AIC and BIC, the smaller the AIC or BIC the better the model

Did the authors assess any correlation in variables? Some variables could have multicolinearity

Ans: We reran the model and checked for multicolinearity and all variables in the model had a variance inflation factor of less than 5, therefore we are not worried about multicolinearity. Check page 13.

Geospatial Analysis

To examine the clustering and spatial distribution of neonatal mortality across provinces in Zambia, this study utilized Quantum Geographic Information System (QGIS) version 3.34.1 to construct a univariate choropleth map. Spatial analysis was conducted at the provincial level, with each neonate matched to their provincial residence using geo-coordinate data collected from the Demographic and Health Survey (DHS). The DHS employs pre-defined data assigning each case to a province. Spatial analysis units were defined as clusters of sample households as designated by the Zambian Demographic and Health Survey (ZDHS). The coordinate system utilized was the World Geographic System (WGS) 1984 Universal Transverse Mercator (UTM) Zone 36S.

Not much analysis here; I recommend saying mapping of neonatal mortality was done in QGIS….

Ans: corrected see page 6

Results.

Furthermore, mortality rates were significantly elevated among non-HIV-infected mothers lacking formal education (40.78%), compared to 54.56% among HIV-infected mothers

Statement doesn't make sense at all

Ans: corrected see page 7

Overall, neonatal mortality stood at 41.13% among mothers (both HIV and non-HIV-infected) with no formal education.

This is a summary and should come in a table or initially when giving results

Ans: the table is below with this summary see page 9

The neonatal mortality rate was high at 37.17% among neonates whose mothers (both HIV infected and non-infected mothers) were reported to be working.

What makes 37.17% high in comparison to what?

Corrected see page 8

The so called geospatial analysis can be a table. Also, what's the rationale for the classes in the map? And what makes >57.87 high?

Ans: **We opted to visualize the distribution of neonatal mortality rates on a map because it provides an intuitive way to communicate regional disparities, highlighting which provinces perform better or worse in terms of neonatal outcomes. Comparing percentages between provinces was more effective visually, as the map clearly shows where neonatal mortality is higher relative to other areas. This justified our use of the term "higher" in the context of regional comparison.**

Regarding the rationale for the classes on the map, they were selected based on natural breaks in the data. This method ensures that the provinces are grouped meaningfully by their mortality rates, avoiding arbitrary cutoff points. The threshold of >57.87% reflects the upper range of neonatal mortality rates observed, marking the provinces with the most concerning outcomes. This classification helps draw attention to areas that may need prioritized interventions.

The results of mixed effects model do not show what the random effects were and what the fixed effects were

Ans: Been shown see page 5
