# Supplementary material for: Spatial variations and predictors of neonatal mortality among births to HIV-infected and non-infected mothers in rural Zambia
Source: PLOS Glob Public Health. 2025 Jul 1;5(7):e0003331. doi: 10.1371/journal.pgph.0003331 (PMC12212519; doi:10.1371/journal.pgph.0003331)
Supplement: Attachment — Submitted filename: review report.docx [file pgph.0003331.s001.docx]

The authors attempt to provide an understanding of geospatial variability in HIV cases and deaths in Zambia and also understand the most significant predictors. The study lacks flow and the methods are poorly written. Significant improvements are needed to clarify the key message and the purpose of the study. The title is overly complex and would benefit from reorientation based on clearly defined objectives, results, and conclusions. The authors often repeat statements already presented in the tables. For instance, the discussion on geospatial patterns and predictors of neonatal mortality implies that geospatial mapping is a key component, which is not adequately reflected in the study itself. There are also a lot of repetitive words and sentences and authors can benefit from a revision that enhances readability. For example, Authors keep referring to children – amend to neonates

**Title**: Too crowded and not clear enough.

**Abstract:**

Needs to be more concise and is poorly written. For example, the first two sentences in the background below don’t belong in abstract.

“*Neonatal mortality is the death of a live-born infant within the first 28 completed days of life. Neonatal mortality remains a major public health concern in most African countries, with the Sub-Saharan region being the highest contributor at 27 deaths per 1000 live births, accounting for 43% of the total number of deaths*.”

**Methods**

More specifically the methods section lacks clarity on why spefic variables were chosen, conceptually how they affect the outcomes, the rationale for the methods including choice of mixed effects model.

**On Community level variables;** *Community-level variables in this study were derived by aggregating individual-level data into clusters and incorporated community poverty, community education, community knowledge of family planning (FP) methods, and place of residence.*

Any reason why this was done and why important it is to generalise assessment of range/ICC?

***Data Analysis***

*For descriptive purposes, frequencies and percentages were computed for categorical variables. To determine the association between the outcome variable (neonatal mortality) and the categorical variables, the Uncorrelated Design Based Chi-square test (Rao – Scott Chi-square test) was used. The multilevel mixed-effect logistic regression model was employed to determine the factors affecting newborn mortality. Additionally, the study used an investigator-led approach, all variables were selected from a wide range of literature. The Intra-Class Correlation (ICC), Akaike Information Criteria (AIC), and the Bayesian Information Criteria (BIC) were sufficiently explored to select the best-fit model. Stata version 14.2 was used for analysis.*

Not clear what analysis was done. The authors alude they did AIC and BIC to perform model selection; but was the model/variable (at this point it is very confusing as to what the paper is all about) selection fit into a final model? And why was it done?

In description of Multilevel logistic regression, was it a multivariable model? what were the levels in the hierarchy and what were the fixed effects, what were the random effects? Below is an example

In the description of the Multilevel Logistic Regression model, the following points need to be addressed for clarity:

**Multivariable Model: Confirm if the model included multiple predictors**.

- Levels in the Hierarchy: Clearly define the hierarchical structure:

- Level 1: Individual level (e.g., neonates).

- Level 2: Cluster level (e.g., health facilities or geographical regions).

**Fixed Effects: Specify the predictors considered as fixed effects, such as**:

- Socio-demographic factors (e.g., maternal age, education level, household income).

- Health-related variables (e.g., prenatal care visits, birth weight, delivery method).

**Random Effects: Identify the random effects, which account for variability at the cluster level**:

- Random intercepts (e.g., differences in neonatal mortality rates across clusters).

- Random slopes for some predictors, if applicable (e.g., varying effects of prenatal care visits across different regions).

Providing this detailed information will enhance the clarity and comprehensiveness of the methods section.

What was the outcome and how were the variables in the final model(s) chosen?

Did the authors assess any correlation in variables? Some variables could have multicollinearity

*Geospatial Analysis*

*To examine the clustering and spatial distribution of neonatal mortality across provinces in Zambia, this study utilized Quantum Geographic Information System (QGIS) version 3.34.1 to construct a univariate choropleth map. Spatial analysis was conducted at the provincial level, with each neonate matched to their provincial residence using geo-coordinate data collected from the Demographic and Health Survey (DHS). The DHS employs pre-defined data assigning each case to a province. Spatial analysis units were defined as clusters of sample households as designated by the Zambian Demographic and Health Survey (ZDHS). The coordinate system utilized was the World Geographic System (WGS) 1984 Universal Transverse Mercator (UTM) Zone 36S.*

Not much analysis here; I recommend saying mapping of neonatal mortality was done in QGIS….

**Results.**

*Furthermore, mortality rates were significantly elevated among non-HIV-infected mothers lacking formal education (40.78%), compared to 54.56% among HIV-infected mothers*

Statement doesn’t make sense at all

*Overall, neonatal mortality stood at 41.13% among mothers (both HIV and non-HIV-infected) with no formal education.*

This is a summary and should come in a table or initially when giving results

*The neonatal mortality rate was high at 37.17% among neonates whose mothers (both HIV infected and non-infected mothers) were reported to be working.*

What makes 37.17% high in comparison to what?

The so called geospatial analysis can be a table. Also, whats the rationalled for the classes in the map? And what makes >57.87 high?

The results of mixed effects model do not show what were the random effects and what were the fixed effects
