# Supplementary material for: Spatial variations and predictors of neonatal mortality among births to HIV-infected and non-infected mothers in rural Zambia
Source: PLOS Glob Public Health. 2025 Jul 1;5(7):e0003331. doi: 10.1371/journal.pgph.0003331 (PMC12212519; doi:10.1371/journal.pgph.0003331)
Supplement: Attachment — Submitted filename: Response to reviewers.docx [file pgph.0003331.s004.docx]

*PLoS Global Public Health*

Dear Editor,

We appreciate the insightful comments and constructive feedback provided by the reviewers on our manuscript, *"Spatial Variations and Predictors of Neonatal Mortality among HIV-Infected and Non-Infected Mothers in Rural Zambia."* Their observations have significantly improved the quality of our study. Below, we present a point-by-point response detailing how we have addressed each concern.

**Reviewer 1**

The authors attempt to provide an understanding of geospatial variability in HIV cases and deaths in Zambia and also understand the most significant predictors. The study lacks flow and the methods are poorly written. Significant improvements are needed to clarify the key message and the purpose of the study. The title is overly complex and would benefit from reorientation based on clearly defined objectives, results, and conclusions. The authors often repeat statements already presented in the tables. For instance, the discussion on geospatial patterns and predictors of neonatal mortality implies that geospatial mapping is a key component, which is not adequately reflected in the study itself. There are also a lot of repetitive words and sentences and authors can benefit from a revision that enhances readability. For example, Authors keep referring to children – amend to neonates

**Ans: Corrected**

**Title**: Too crowded and not clear enough.

**Ans: Refined (see page 1)**

**Abstract:**

Needs to be more concise and is poorly written. For example, the first two sentences in the background below don’t belong in abstract.

“*Neonatal mortality is the death of a live-born infant within the first 28 completed days of life. Neonatal mortality remains a major public health concern in most African countries, with the Sub-Saharan region being the highest contributor at 27 deaths per 1000 live births, accounting for 43% of the total number of deaths*.”

**Ans: Refined (see page 2)**

**Methods**

More specifically the methods section lacks clarity on why spefic variables were chosen, conceptually how they affect the outcomes, the rationale for the methods including choice of mixed effects model.

**Ans: Done, an explanation has been included in the Dependent and independent variables**

**Section see page 4. Also we have added a conceptual framework to support where we obtained the variables from.**

**On Community level variables;** *Community-level variables in this study were derived by aggregating individual-level data into clusters and incorporated community poverty, community education, community knowledge of family planning (FP) methods, and place of residence.*

**Ans: We have actually removed them because we didn’t have enough power to actually have more variables in the model.**

***Data Analysis***

*For descriptive purposes, frequencies and percentages were computed for categorical variables. To determine the association between the outcome variable (neonatal mortality) and the categorical variables, the Uncorrelated Design Based Chi-square test (Rao – Scott Chi-square test) was used. The multilevel mixed-effect logistic regression model was employed to determine the factors affecting newborn mortality. Additionally, the study used an investigator-led approach, all variables were selected from a wide range of literature. The Intra-Class Correlation (ICC), Akaike Information Criteria (AIC), and the Bayesian Information Criteria (BIC) were sufficiently explored to select the best-fit model. Stata version 14.2 was used for analysis.*

Not clear what analysis was done. The authors alude they did AIC and BIC to perform model selection; but was the model/variable (at this point it is very confusing as to what the paper is all about) selection fit into a final model? And why was it done?

**Ans: What Analysis Was Done?**

- **Descriptive Analysis: Frequencies and percentages were computed for categorical variables to provide a summary of the data.**
- **Association Testing: The Rao–Scott Chi-square test was used to determine the association between neonatal mortality (the outcome) and categorical variables, accounting for the survey design.**
- **Multilevel Mixed-Effects Logistic Regression: This model was employed to analyze the factors influencing neonatal mortality, accounting for individual-level and community-level variations. The mixed-effects model is appropriate because the dataset has a hierarchical structure (individuals nested within communities).**

**We used the Akaike Information Criterion (AIC) and Bayesian Information Criterion (BIC) for model selection to identify the most parsimonious model that balances goodness of fit with complexity. AIC rewards goodness of fit but penalizes the number of parameters, while BIC imposes a stronger penalty for model complexity. These criteria help us ensure the final model avoids overfitting by retaining only variables that meaningfully contribute to explaining neonatal mortality. This process ensures robust results aligned with the study’s focus on key predictors.**

In description of Multilevel logistic regression, was it a multivariable model? what were the levels in the hierarchy and what were the fixed effects, what were the random effects? Below is an example

In the description of the Multilevel Logistic Regression model, the following points need to be addressed for clarity:

**Multivariable Model: Confirm if the model included multiple predictors**.

- Levels in the Hierarchy: Clearly define the hierarchical structure:

- Level 1: Individual level (e.g., neonates).

- Level 2: Cluster level (e.g., health facilities or geographical regions).

**Ans: Yes, the multivariable model did include multiple predictors to explore the factors associated with neonatal mortality. These predictors spanned individual characteristics such as maternal age, education, and birthweight, along with cluster-level factors like place of delivery and regional location.**

**Regarding the hierarchical structure, we used a multilevel model to account for the nested nature of the data:**

- **Level 1: Individual-level predictors (e.g., characteristics of neonates and their mothers)**
- **Level 2: Cluster-level variables (e.g., geographical regions or enumeration areas)**

**Fixed Effects: Specify the predictors considered as fixed effects, such as**:

- Socio-demographic factors (e.g., maternal age, education level, household income).

- Health-related variables (e.g., prenatal care visits, birth weight, delivery method).

**Ans: Noted they have been specified see page 4**

**Random Effects: Identify the random effects, which account for variability at the cluster level**:

- Random intercepts (e.g., differences in neonatal mortality rates across clusters).

- Random slopes for some predictors, if applicable (e.g., varying effects of prenatal care visits across different regions).

Providing this detailed information will enhance the clarity and comprehensiveness of the methods section.

**Ans: Been provided check on page page 13**

What was the outcome and how were the variables in the final model(s) chosen?

**Ans: the outcome variable in the model was neonatal mortality coded as dead or alive. The best fit model was selected through the AIC and BIC, the smaller the AIC or BIC the better the model**

Did the authors assess any correlation in variables? Some variables could have multicolinearity

**Ans: We reran the model and checked for multicolinearity and all variables in the model had a variance inflation factor of less than 5, therefore we are not worried about multicolinearity. Check page 13.**

*Geospatial Analysis*

*To examine the clustering and spatial distribution of neonatal mortality across provinces in Zambia, this study utilized Quantum Geographic Information System (QGIS) version 3.34.1 to construct a univariate choropleth map. Spatial analysis was conducted at the provincial level, with each neonate matched to their provincial residence using geo-coordinate data collected from the Demographic and Health Survey (DHS). The DHS employs pre-defined data assigning each case to a province. Spatial analysis units were defined as clusters of sample households as designated by the Zambian Demographic and Health Survey (ZDHS). The coordinate system utilized was the World Geographic System (WGS) 1984 Universal Transverse Mercator (UTM) Zone 36S.*

Not much analysis here; I recommend saying mapping of neonatal mortality was done in QGIS….

**Ans: corrected see page 6**

**Results.**

*Furthermore, mortality rates were significantly elevated among non-HIV-infected mothers lacking formal education (40.78%), compared to 54.56% among HIV-infected mothers*

Statement doesn’t make sense at all

**Ans: corrected see page 7**

*Overall, neonatal mortality stood at 41.13% among mothers (both HIV and non-HIV-infected) with no formal education.*

This is a summary and should come in a table or initially when giving results

**Ans: the table is below with this summary see page 9**

*The neonatal mortality rate was high at 37.17% among neonates whose mothers (both HIV infected and non-infected mothers) were reported to be working.*

What makes 37.17% high in comparison to what?

**Corrected see page 8**

The so called geospatial analysis can be a table. Also, what’s the rationale for the classes in the map? And what makes >57.87 high?

Ans: **We opted to visualize the distribution of neonatal mortality rates on a map because it provides an intuitive way to communicate regional disparities, highlighting which provinces perform better or worse in terms of neonatal outcomes. Comparing percentages between provinces was more effective visually, as the map clearly shows where neonatal mortality is higher relative to other areas. This justified our use of the term "higher" in the context of regional comparison.**

**Regarding the rationale for the classes on the map, they were selected based on natural breaks in the data. This method ensures that the provinces are grouped meaningfully by their mortality rates, avoiding arbitrary cutoff points. The threshold of >57.87% reflects the upper range of neonatal mortality rates observed, marking the provinces with the most concerning outcomes. This classification helps draw attention to areas that may need prioritized interventions.**

The results of mixed effects model do not show what the random effects were and what the fixed effects were

**Ans: Been shown see page 5**
