# Supplementary material for: Spatial variations and predictors of neonatal mortality among births to HIV-infected and non-infected mothers in rural Zambia
Source: PLOS Glob Public Health. 2025 Jul 1;5(7):e0003331. doi: 10.1371/journal.pgph.0003331 (PMC12212519; doi:10.1371/journal.pgph.0003331)
Supplement: Attachment — Submitted filename: Response_to_Reviewers_auresp_2.docx [file pgph.0003331.s005.docx]

Samson Shumba

The University of Zambia,

[Samsonshumba1@gmail.com](mailto:Samsonshumba1@gmail.com).

13^th^ April, 2025

**Editorial Team**
*PLOS Global Public Health*

Dear Editors,

**Re: Response to Reviewer Comments – Manuscript Title: “Spatial Variations and Predictors of Neonatal Mortality among HIV-Infected and Non-Infected Mothers in Rural Zambia”**

We sincerely thank you and the reviewers for the insightful feedback provided on our manuscript. We have addressed all the comments and made the necessary revisions. Below is a summary of our responses to each reviewer’s concern:

**Reviewer Comment 1:**
*"The use of 'geospatial variations' is misleading, as the methods section does not describe an appropriate analytical approach to support this claim. The maps presented do not effectively communicate a clear message beyond displaying different color codes, and the statistical significance of the observed patterns is unclear. The authors should consider using Moran’s I to identify spatial clusters of areas with similar risks for the outcome of interest."*

**Response:**

- Moran’s I spatial autocorrelation analysis was conducted using R version 4.3.2 with relevant spatial packages including spdep, sf, tmap, and rgdal.
- The methods and results sections now describe the procedure and significance testing of Moran’s I in detail.

**Reviewer Comment 2:**
*"Regarding the multilevel modeling approach, it is standard practice to run three models... The current analysis does not follow this structured approach, and the methodology should be revised accordingly."*

**Response:**

- We restructured the multilevel analysis to follow standard practices:
  - Model 0 (Null Model): Includes only the response variable and random effects
  - Model 1: Includes individual-level predictors
  - Model 3: Includes both individual and contextual-level predictors with random effects

**Reviewer Comment 3:**
*"The interpretation of odds ratios is incorrect and requires a clearer and more structured explanation."*

**Response:**

- We revised the interpretation of odds ratios to ensure accuracy.

**Reviewer Comment 4:**
*"Finally, the manuscript contains several grammatical errors, and a thorough review and revision for language clarity would be helpful."*

**Response:**

- The manuscript was thoroughly reviewed for grammar and language clarity.
- Sentences were revised to enhance flow, precision, and overall readability.

We have attached both a clean version of the revised manuscript and a version with tracked changes. We intend to formally submit the revised manuscript by 17:00 tomorrow, pending any additional feedback.

Thank you once again for your valuable comments and for the opportunity to strengthen our work.

Sincerely,
**Samson Shumba**
On behalf of all co-authors
*Corresponding Author*
