# Supplementary material for: Spatial variations and predictors of neonatal mortality among births to HIV-infected and non-infected mothers in rural Zambia
Source: PLOS Glob Public Health. 2025 Jul 1;5(7):e0003331. doi: 10.1371/journal.pgph.0003331 (PMC12212519; doi:10.1371/journal.pgph.0003331)
Supplement: Attachment — Submitted filename: Response_to_Reviewers_auresp_3.docx [file pgph.0003331.s006.docx]

**Manuscript ID**: PGPH-D-24-01165R2
**Title**: Spatial Variations and Predictors of Neonatal Mortality among Births to HIV-Infected and Non-Infected Mothers in Rural Zambia

Dear Dr. Odhiambo and Review Team,

We thank you for the opportunity to revise our manuscript and appreciate the constructive comments provided by the editor and reviewers. We have carefully addressed all the suggestions to improve the clarity, accuracy, and overall quality of the manuscript. Below, we provide a point-by-point response to the comments received.

**Editorial Comment 1: Study Objective Clarity**

*“The stated study objective, 'geospatial patterns and predictors of neonatal mortality among both HIV-infected and non-infected mothers in rural Zambia,' suggests that the unit of analysis is the mother. However, the analysis appears to focus on neonatal outcomes. I recommend rewording the objective for clarity and consistency with the analysis.”*

**Response**:
We agree with the editorial observation and have revised the study objective throughout the manuscript to reflect that the unit of analysis is the **neonate** rather than the mother. The revised objective now reads:

**"To examine the spatial variations and predictors of neonatal mortality among births to HIV-infected and non-infected mothers in rural Zambia."**
This update has been made in the Abstract, Introduction, and Methods sections.

**Editorial Comment 2: Clarification of Geospatial Unit of Analysis**

*“I recommend clarifying early in the manuscript whether the primary geospatial unit of analysis is the cluster, subnational area, or both...”*

**Response**:
We have clarified that the primary geospatial unit of analysis for statistical testing was the **DHS cluster**, while **mapping and visualization** were performed at the **subnational level (provincial)** to enhance interpretability for policy use. The Methods section now includes the following clarification:

*“For spatial autocorrelation analysis, the proportions were imported into R version 4.3.2 and analyzed at the DHS cluster level using Moran’s I statistic.”*

**Justification for Using Two Spatial Levels:**

We analyzed spatial autocorrelation at the **DHS cluster level** because clusters are the smallest available geographic units in the dataset. This allowed us to detect local patterns and spatial relationships in neonatal mortality more accurately using Moran’s I statistic.

For the maps, we used **provincial-level data** by aggregating the proportions. This was done to make the results easier to understand and more useful for policymakers, since provinces are commonly used in planning and decision-making. Using both cluster-level analysis and provincial-level maps helped us combine detailed analysis with practical interpretation.

**Additional Edits and Improvements**

- We updated the manuscript title to more accurately reflect the neonatal-level outcome:

**"Spatial Variation and Predictors of Neonatal Mortality among Births to HIV-Infected and Non-Infected Mothers in Rural Zambia."**

- The term **“geospatial patterns”** has been replaced with **“spatial variation”** where appropriate to better reflect the nature of the analysis and visualization.
- We verified and corrected all references, ensuring none were retracted and all citations are accurate.

We believe these revisions address the concerns raised and significantly strengthen the manuscript. We sincerely thank the editorial team for the insightful feedback and the opportunity to improve our work.

Kind regards,
**Samson Shumba**
**Corresponding Author**
